# Protocol for Converting DICOM Files to STL Models Using 3D Slicer and Ultimaker Cura

**DOI:** 10.3390/jpm15030118

**Published:** 2025-03-19

**Authors:** Malena Pérez-Sevilla, Fernando Rivas-Navazo, Pedro Latorre-Carmona, Darío Fernández-Zoppino

**Affiliations:** 1 Department of Digitalization, Universidad de Burgos, Avda. Cantabria sn, 09006 Burgos, Spain; mpsevilla@ubu.es; 2 Department of Electromechanical Engineering, Universidad de Burgos, Avda. Cantabria sn, 09006 Burgos, Spain; frivas@ubu.es; 3 Departamento de Ingeniería Informática, Universidad de Burgos, Avda. Cantabria sn, 09006 Burgos, Spain; 4 Facultad de Ciencias de la Salud, Universidad de Burgos, Paseo de los Comendadores sn, 09001 Burgos, Spain; dfx@ubu.es

**Keywords:** DICOM-to-STL conversion, 3D Slicer and Ultimaker Cura, three-dimensional anatomical modelling, medical image processing, surgical planning, Rapid Prototyping in Medicine

## Abstract

**Background/Objectives**: 3D printing has become an invaluable tool in medicine, enabling the creation of precise anatomical models for surgical planning and medical education. This study presents a comprehensive protocol for converting DICOM files into three-dimensional models and their subsequent transformation into GCODE files ready for 3D printing. **Methods**: We employed the open-source software “3D Slicer” for the initial conversion of the DICOM files, capitalising on its robust capabilities in segmentation and medical image processing. An optimised workflow was developed for the precise and efficient conversion of medical images into STL models, ensuring high fidelity in anatomical structures. The protocol was validated through three case studies, achieving elevated structural fidelity based on deviation analysis between the STL models and the original DICOM data. Furthermore, the segmentation process preserved morphological accuracy within a narrow deviation range, ensuring the reliable replication of anatomical features for medical applications. Our protocol provides an effective and accessible approach to generating 3D anatomical models with enhanced accuracy and reproducibility. In later stages, we utilised the “Ultimaker Cura” software to generate customised GCODE files tailored to the specifications of the 3D printer. **Results**: Our protocol offers an effective, accessible, and more accurate solution for creating 3D anatomical models from DICOM images. Furthermore, the versatility of this approach allows for its adaptation to various 3D printers and materials, expanding its utility in the medical and scientific community. **Conclusions**: This study presents a robust and reproducible approach for converting medical data into physical three-dimensional objects, paving the way for a wide range of applications in personalised medicine and advanced clinical practice. The selection of sample datasets from the 3D Slicer repository ensures standardisation and reproducibility, allowing for independent validation of the proposed workflow without ethical or logistical constraints related to patient data access. However, we acknowledge that future work could expand upon this by incorporating real patient datasets and benchmarking the protocol against alternative segmentation methods and software packages to further assess performance across different clinical scenarios. Essentially, this protocol can be particularly characterised by its commitment to open-source software and low-cost solutions, making advanced 3D modelling accessible to a wider audience. By leveraging open-access tools such as “3D Slicer” and “Ultimaker Cura”, we democratise the creation of anatomical models, ensuring that institutions with limited resources can also benefit from this technology, promoting innovation and inclusivity in medical sciences and education.

## 1. Introduction

Over recent decades, additive manufacturing, more commonly referred to as three-dimensional (3D) printing, has rapidly transitioned from an experimental technique into a revolutionary technology with a significant impact across numerous disciplines, including engineering, manufacturing, and particularly medicine [1,2]. Within the medical arena, the transformative potential of 3D printing has already demonstrated profound implications, from precision surgical planning and custom prosthetics to enhanced medical education through patient-specific anatomical modelling [3,4], specifically for medical applications [5]. The capability to fabricate tangible anatomical replicas derived from diagnostic medical imaging techniques—such as Computed Tomography (CT) and Magnetic Resonance Imaging (MRI)—allows clinicians and researchers to not only visualise complex structures but also to rehearse intricate surgical procedures and improve clinical outcomes through meticulous preoperative planning [6,7].

Central to this transformational application of 3D printing is the accurate conversion of digital imaging data, primarily encoded in the standardised Digital Imaging and Communications in Medicine (DICOM) format, into precise three-dimensional anatomical models in the widely recognised Stereolithography (STL) format. This conversion process, while conceptually straightforward, is fraught with practical challenges, including inconsistencies in segmentation accuracy, degradation or loss of fine anatomical detail, and significant variability attributable to differences in operator proficiency, imaging quality, and software algorithms [7,8]. Additionally, existing methodologies often rely on proprietary software solutions characterised by high licensing fees and steep learning curves, considerably limiting their adoption in resource-constrained clinical settings [6,8].

Acknowledging these inherent challenges, this study presents a meticulously developed, robust, and thoroughly validated open-source protocol for the precise and efficient transformation of DICOM images into high-fidelity STL models suitable for 3D printing. Central to this protocol are the open-source platforms 3D Slicer and Ultimaker Cura, selected specifically for their accessibility, advanced image processing capabilities, and versatility within diverse healthcare [9]. Our approach strategically integrates both automated and semi-automated segmentation algorithms tailored to tissue density and printing applications, addressing common anatomical scenarios including bone structures, soft tissues, tumours, and complex multi-tissue interfaces [6]. This targeted adaptation ensures enhanced morphological accuracy and reproducibility, crucial in clinical and educational settings where anatomical fidelity is paramount.

To rigorously ensure structural fidelity, our protocol incorporates systematic evaluations of model accuracy through quantitative deviation analyses. Metrics such as the Dice Similarity Coefficient (DSC), volumetric superposition, and Hausdorff distance comparisons are employed to rigorously quantify deviations between generated STL models and the original DICOM datasets, affirming the high morphological accuracy and clinical utility of the models [5,7]. This rigorous verification step differentiates our workflow from many existing methods, which typically rely on subjective qualitative assessments or do not systematically document fidelity criteria, potentially compromising reliability in sensitive medical contexts [6].

Moreover, our protocol significantly optimises the DICOM-to-STL conversion process, achieving a considerable reduction in processing time—approximately 30%—compared with conventional manual segmentation methods. This efficiency is accomplished without sacrificing anatomical accuracy, making it especially appealing in scenarios that demand rapid model generation, such as surgical planning or intraoperative decision-making contexts [10]. Furthermore, the implementation of this protocol exclusively utilises open-source software, 3D Slicer and Ultimaker Cura, thus eliminating the significant economic barriers associated with commercial software and enabling broad access to advanced 3D anatomical modelling capabilities within economically diverse medical and academic environments [8,11].

Through the validation of this protocol across diverse anatomical case studies, including spinal vertebrae, mandibular structures derived from Cone Beam Computed Tomography (CBCT), and intracranial brain tumours visualised via Magnetic Resonance Imaging (MRI) [9], the versatility, robustness, and reliability of the proposed workflow have been extensively demonstrated. Moreover, the employment of widely adopted open-source tools such as 3D Slicer and Ultimaker Cura underscores a broader commitment to democratising advanced anatomical modelling technology, making high-fidelity medical models widely accessible beyond high-resource medical centres, thereby fostering inclusivity and innovation in healthcare and medical education [12].

Looking ahead, this work lays the foundation for further advancements, particularly through the potential integration of artificial intelligence (AI)-enhanced segmentation techniques [12]. Future expansions could leverage AI-driven segmentation processes, promising increased automation, improved reproducibility, and heightened precision—critical when differentiating complex anatomical structures, such as intricate dental anatomies, cranial-maxillofacial complexities, and tumour boundaries [13]. Additionally, exploring emerging capabilities in multi-material and multi-colour 3D printing technologies represents a promising avenue for providing a richer anatomical context and the enhanced clinical interpretability of printed models, thus further supporting the evolution of personalised medicine [14].

In summary, this study not only proposes a technically rigorous and economically accessible pathway for converting medical images into highly accurate physical models but also significantly advances the field of personalised medical practice. By democratising advanced 3D printing capabilities through open-source technologies and clearly outlining validated and reproducible methodologies, we contribute decisively to reducing technological and economic barriers, ultimately improving clinical outcomes, fostering advanced educational practices, and driving innovation in personalised medicine on a global scale.

## 2. Protocol Description

Having explored the extensive potential of 3D printing in fields such as medicine, engineering, and science, and highlighting the crucial ability to transform medical images into personalised three-dimensional anatomical models, we delve into the practical core of our proposal. The transition from theory to practical application is made through a detailed protocol that guides the conversion of DICOM files to 3D STL models [15].

This process not only represents a bridge between digital information and its physical manifestation but also underlines the importance of specific tools such as 3D Slicer and Ultimaker Cura in carrying out this work. The proposed protocol is aimed at making it easier to create anatomical models with high structural fidelity and direct applicability in clinical practice, biomedical research, and advanced medical education.

The conversion of DICOM files to three-dimensional STL models and their subsequent transformation to GCODE files ready for 3D printing constitutes a fundamental process in the manufacture of accurate anatomical models for medical applications. This detailed protocol has been designed to facilitate the creation of three-dimensional anatomical models with high structural fidelity, taking advantage of the advanced capabilities of the open-source 3D Slicer and Ultimaker Cura software. The proposed methodology not only ensures the efficient and accurate conversion of medical data into three-dimensional physical models but also allows for the customisation of the models to meet the specific needs of each medical application, from surgical planning to medical education.

The protocol begins with the initialisation of the 3D Slicer software (Version 5.2.2) for importing and processing DICOM files, followed by a series of meticulously designed steps for segmentation and volume rendering, ensuring the anatomical integrity of the STL models. This process includes configuring views, activating specific modules for volume rendering and segmentation, and applying advanced tools for segment editing and smoothing. The conversion from STL to GCODE using Ultimaker Cura (Version 5.9) represents the final phase of the protocol, where models are prepared for 3D printing, adjusting specific printing settings to ensure optimal results.

**Figure 1:**
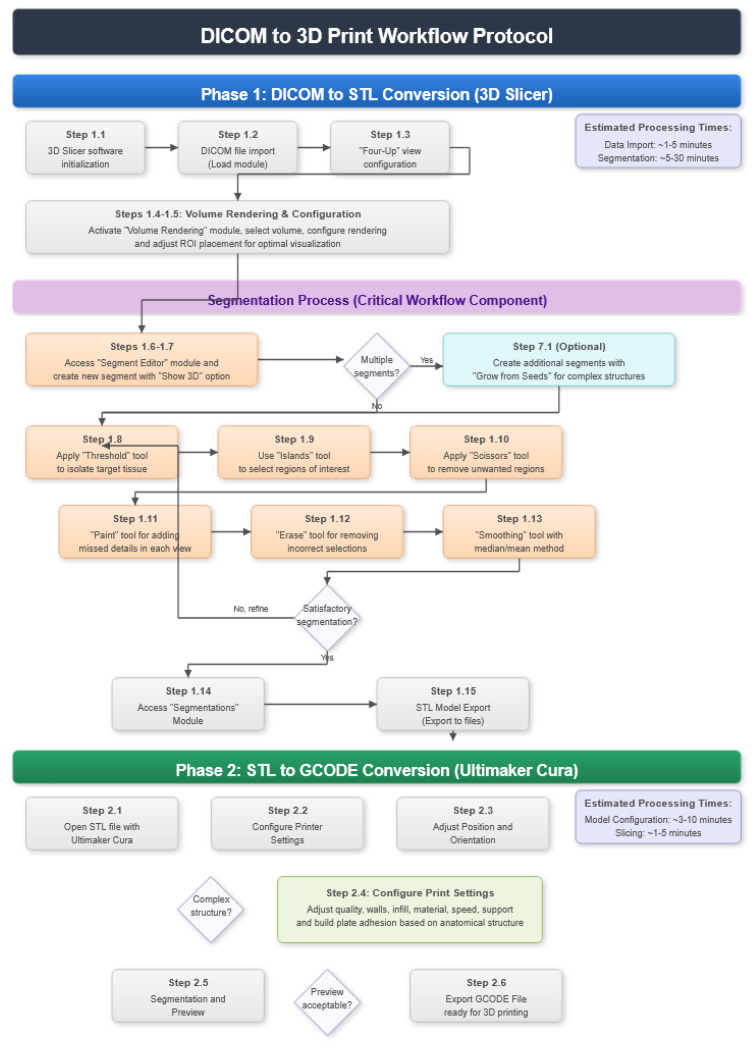
Flow diagram summarising the DICOM to GCODE file conversion process.

The specific steps of the protocol are detailed below, and a flow chart is shown in Figure 1. It can be organised into two main phases: the conversion from DICOM to STL and from STL to GCODE. Each phase is designed to maximise the precision, efficiency, and customisation of the final model, allowing for the creation of complex anatomical models with materials that are safe for medical use. This protocol is particularly useful for its accessibility and versatility, offering an effective solution for implementation in various clinical and educational contexts.

### 2.1. DICOM-to-STL File Conversion

For optimal performance, 3D Slicer and Ultimaker Cura benefit from a system with at least a quad-core CPU (e.g., Intel i5 or AMD Ryzen 5), 16 GB of RAM, and a dedicated GPU (for rendering-intensive tasks). Large DICOM datasets, particularly those from high-resolution MRI or CT scans, may require additional memory and storage capacity. Users experiencing slow performance can optimise segmentation by reducing the voxel resolution, enabling GPU acceleration, and adjusting memory cache settings within 3D Slicer. The particular steps and details of the protocol are as follows:

**STEP 1**: 3D Slicer software initialisation

**STEP 2**: DICOM file import

- At the DICOM module, select “Load” in order to import the corresponding DICOM file for the medical image of interest.

**STEP 3**: “Four-Up” view configuration

- Go to the “Layout Selection” in the tool bar, and select “Four-Up” view. This configuration allows for the visualisation of DICOM images in three different views, along the 3D view.

**STEP 4**: “Volume Rendering” Module activation

- Select the “Volume Rendering” module in the toolbar.

Print settings must be adjusted according to the anatomical structure and intended application. While universal values are difficult to establish due to variations in printer models and materials, general recommendations include (a) Bone structures (e.g., vertebrae, jawbone): Infill 20–40%, wall thickness ≥1.2 mm, supports enabled for complex geometries; (b) Soft tissues or tumour models: Infill 10–20%, flexible filament recommended for better simulation, supports enabled; (c) Vascular or thin-walled structures: Infill 5–15%, increased wall thickness ≥1.5 mm, supports essential. These values serve as a baseline and should be fine-tuned based on clinical needs and material constraints.

**STEP 5**: Volume selection and rendering configuration

- Select the volume of interest in the drop-down menu, and activate the rendering function.
- Configure the rendering process by accessing the “Display” drop-down. Choose the most appropriate “preset” option in terms of the image and anatomical model tissue.
- Use the Shift function to adjust the rendered visible tissues, and enable the “Crop” option in order to cut the “region of interest”.
- Make the Region of Interest (ROI) display visible in order to visualise it, and adjust the ROI volume rendering square in the region of interest for the 3D view.

**STEP 6**: “Segment Editor” Module [16,17] access

- Go to the “Segment Editor” module in the toolbar.

**STEP 7**: Generation of a new Segment and Visualisation

- Select “Add new empty segment”, and assign a descriptive name to the segment.
- Press “Show 3D” to visualise the segment in the corresponding 3D View.

**STEP 7.1**: Generation of multiple segments (Optional) Whenever several specific structures or regions of interest in medical images are to be segmented, we might opt for the creation of additional segments. The following steps apply in case of multiple segmentations:

- Creation of new segments: Start the creation of additional segments for the corresponding structures.
- “Grow from Seeds” tool: Select the “Grow from Seeds” tool for a more detailed segmentation.
- Seed placement: Identify and select reference points or seeds inside the regions to be segmented.
- Initiate the segmentation process: Activate the “Grow from Seeds” function, and allow the tool to expand the segmentation from the seeds to the adjacent regions.

This optional step is particularly beneficial when segmenting complex anatomical structures, such as cranial-maxillofacial regions or tumour delineation, where separating multiple tissues improves anatomical fidelity. Multi-segmentation allows for better differentiation between adjacent structures, reducing artefacts in final STL models. However, users should be aware of potential pitfalls such as overlapping segments, incorrect tissue classification, or segmentation gaps, which can impact the integrity of the 3D model. To mitigate these issues, the protocol recommends manual refinement of segment boundaries, the use of seed-based segmentation tools, and careful post-processing in the “Segment Editor” module.

**STEP 8**: “Threshold” tool

- Select “Threshold” tool.
- Adjust the threshold of the Threshold range until it includes the entire region of interest, and apply the settings.

**STEP 9**: Use of the “Islands” tool

- Use the “Islands” tool.
- Select the “Keep selected islands” option, and mark the areas of interest in one of the views.

**STEP 10**: Use of the “Scissors” tool

- Select the “Scissors” tool.
- Configure the options “Erase inside”, “Free-form’,’ and “Unlimited”.
- In the views, select and delete unwanted parts until the desired anatomical structure is obtained.

**STEP 11**: “Paint” tool

- Use the “Paint” tool.
- Paint those parts which have not been selected yet and which form part of the model for each one of the “cuts” of the three views.

**STEP 12**: “Erase” tool

- Use the “Erase” tool.
- Paint the selected parts, which do not form part of the model for each cut in the three views.

**STEP 13**: “Smoothing” tool

- Select the “Smoothing” tool.
- Choose the “median” or “mean” method, and configure the kernel size as needed.

**STEP 14**: Access the “Segmentations” Module

- Go to the “Segmentations” Module in the toolbar.

**STEP 15**: STL Model Export

- Go to “Export/import models and label maps”.
- In the menu, select “model” as the output option.
- In “Export to files”, choose the destination folder.
- Select the STL file format, and, finally, click on “Export”.

### 2.2. STL-to-GCODE File Conversion

**STEP 1**: Open the STL file with Ultimaker Cura

- Launch the Ultimaker Cura program on your computer.
- Open the STL file you want to print from the “Open File” option in the menu.

**STEP 2**: Configure the Printer Settings

- Access the printer settings within Ultimaker Cura.
- Be sure to select the appropriate printer model and material settings for your 3D printer.

**STEP 3**: Adjust the Position and Orientation of the Model

- If necessary, make adjustments to the position, scale, or rotation of the model on the build platform within Ultimaker Cura. This allows you to optimise the layout of the model on the printing platform.

**STEP 4**: Configure Print Settings In the current phase, it is important to adapt the printing parameters so that personal preferences are synchronised with project requirements. Below is an outline of the main configurations.

Quality:

- Adjust the layer height and line width to refine the print quality, considering the balance between print resolution and time efficiency.

Walls:

- The print quality can be improved by increasing the number of walls, which strengthens the structure and improves the surface final result. Adjust the wall line count and interior-to-exterior orientation to optimise for strength and aesthetics.

Upper or Lower:

- Set the number of top and bottom layers, select a pattern for these surfaces, and enable flattening with a specific pattern if necessary. These adjustments influence the surface texture and structural integrity of the printed object.

Infill:

- Determine the density and pattern of the infill, taking into account the trade-off between the weight of the object and its structural robustness. The fill-in sequence in relation to the wall print can also affect the final quality of the print.

Material:

- Set the print temperature, initial layer temperature, final temperature, and build plate temperature according to the filament material specifications to ensure optimal extrusion and adhesion. The flow rate may also need adjustment to achieve the desired extrusion quality. It is crucial to refer to the filament manufacturer’s guidelines for specific settings, as these can vary significantly between different materials and brands.

Speed:

- Control the print speed to balance the print quality and time efficiency. Slower speeds typically result in higher-quality prints by allowing for more precise material deposition.

Travel:

- Enable retraction to prevent dripping and threading by adjusting the distance and speed of retraction based on the printer capabilities and filament used.

Refrigeration:

- Adjust the fan speed to regulate cooling, which can significantly affect the print quality, especially on overhangs and bridges. It is important to note that the optimal cooling configuration will vary depending on the printing material used since each material has different thermal properties that affect its behavior against rapid cooling.

Support:

- Choose the appropriate support structure type and pattern to ensure the successful printing of complex geometries, adjusting the adhesion to the build plate as necessary.

Build Plate Adhesion:

- Select an adhesion type to improve the first coat’s adhesion to the build plate, reducing problems associated with poor first coat adhesion.

Note: Adjusting print settings is a critical step in achieving quality prints. These settings should be tailored based on the specific filament used in your 3D printer, taking into account the filament manufacturer’s recommendations to ensure optimal results.

**STEP 5**: Segmentation

- In order to continue the STL file to GCODE conversion process, select the “Segmentation” option in Ultimaker Cura.
- The segmentation tool provides valuable information such as the estimated model weight and estimated printing time, among other crucial data.
- Once the segmentation is complete, go to the “Preview” option. Here, a sidebar will appear on the right side of the screen, giving you the ability to preview the materialisation process layer by layer, from the base to the top of the model.

**STEP 6**: Export the GCODE File

- Once all the print settings are configured, select the “File” option in the Ultimaker Cura menu.
- Choose the “Export” option, and select “GCODE” as the output file type.
- Save the GCODE file to the desired location for later use in your 3D printer.

## 3. Results and Discussion

We present in this section a practical and critical assessment of the proposed protocol, through several case studies. The transition from the rigorous protocol to the real application and evaluation of the results allows for not only the validation of the effectiveness and precision of the proposed method, but also the exploration of its practical applications in medicine and biomedical research. Through the presentation of specific cases, such as a vertebrae, lower jaw, and brain tumour, this section not only demonstrates the applicability and clinical relevance of the generated 3D models but also delves into the discussion of the challenges, limitations, and the potential for expansion of these technologies in the future.

Several case studies will be presented in Section 3.1, Section 3.2 and Section 3.3. We will first show the vertebrae case, including tools used for the printing segmentation. Nevertheless, for the lower jaw and the brain tumour, only the final results will be shown. These final results come from two protocols:

1. The DICOM-to-STL protocol, using 3D Slicer.
2. The STL-to-GCODE protocol, using Ultimaker Cura.

In Section 3.1.3, Section 3.2.3, and Section 3.3.3, images of printed figures are presented, which have been photographed alongside a calibration cube with dimensions of 15 × 15 × 15 mm. This cube serves as a measurement reference to provide an accurate scale, thus enabling direct size comparison with the printed figures. It is important to bear this reference in mind when viewing the photographs.

The figures depicted constitute an amalgam of selected images grouped according to their mode of display. This methodological approach ensures a coherent interpretation and facilitates the visual comparison of the figures. The use of the calibration cube in all images guarantees scale consistency, which is crucial for validating the dimensions and proportions of the printed figures. The essential details regarding the printing parameters are available in the Supplementary Materials.

For the printing of these figures, two different FDM printers [18] were used: the Artillery Sidewinder X2 [19] and the Creality Ender 3 [20]. It is pertinent to note that, although both printers operate using Fused Deposition Modelling (FDM) technology, they exhibit significant differences in their extrusion systems that impact their capabilities and print quality. Additionally, both printers are equipped with a 0.4 mm nozzle, which is standard for achieving a balance between print speed and detail resolution. This nozzle size plays a crucial role in determining the fineness of the printed figures, allowing for detailed work while maintaining reasonable printing times.

The Artillery Sidewinder X2 is characterised by its direct drive extrusion system (Titan type), which affords it a considerable advantage in terms of compatibility with a wide range of materials. This type of extrusion enhances control over the material flow and reduces the likelihood of clogs, thereby facilitating printing with materials that require higher temperatures or possess more complex properties.

Conversely, the Creality Ender 3 employs an FDM extrusion system (Bowden system), wherein the motor that pushes the filament is separated from the print head. Although this design may contribute to lighter and faster printing, it limits the range of materials that can be effectively used due to the greater distance the material must travel from the extrusion motor to the nozzle. This can affect the precision when extruding more complex or flexible materials. The following key parameters are summarised in Table 1.

### 3.1. A Simple and Common Case

#### 3.1.1. DICOM-to-STL File

Figure 2, Figure 3, Figure 4, Figure 5, Figure 6, Figure 7, Figure 8, Figure 9, Figure 10, Figure 11, Figure 12, Figure 13 and Figure 14 show the complete process. The DICOM file is opened. In this case, the CTChest file is downloaded from the “Download Sample Data” option [21].

Steps 4 to 7 in the protocol are carried out, and once those steps are completed, the “threshold” tool is used.

**Figure 2:**
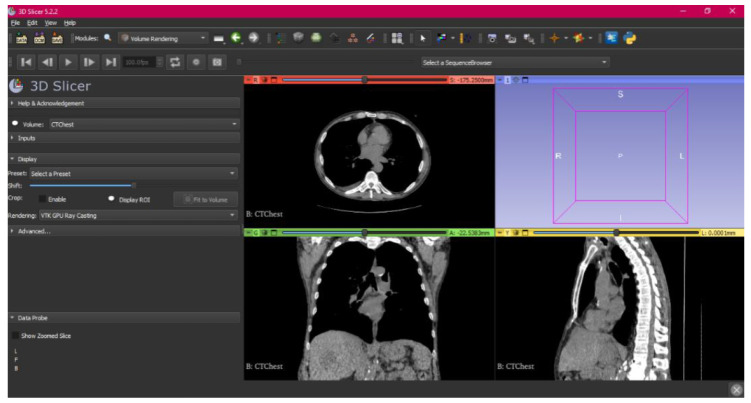
DICOM CTChest Sample Data in “Four-Up” configuration.

**Figure 3:**
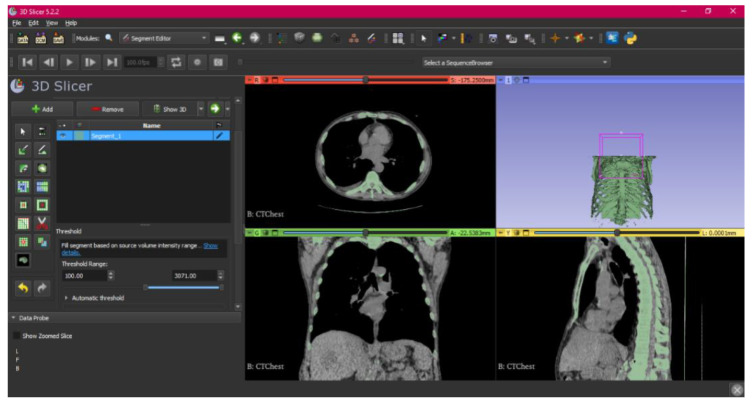
Segment Editor, “Threshold” tool.

When using the tool, the selected parts are visualised in a green colour in the four views. The tool is therefore helping establish the image values as a specific value given by the user if they are below, above, or in between simple values, helping select the osseous part, in this case. Then, the “Islands” tool is used.

**Figure 4:**
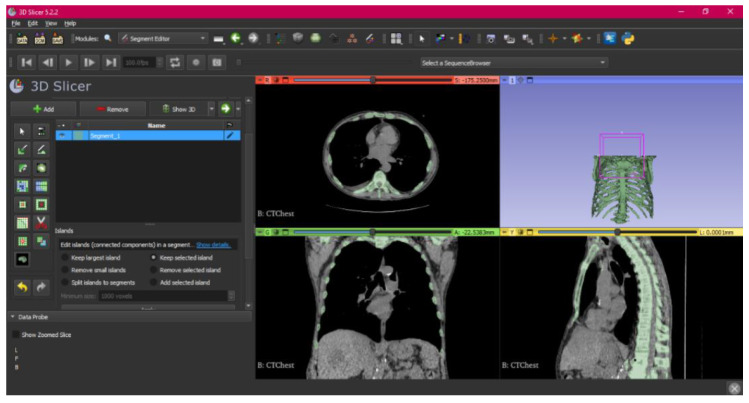
Segment Editor, “Islands” tool.

In this case, the tool has allowed for the selection of connected regions defined as non-empty groups of voxels, which do not touch each other but are surrounded by empty voxels, allowing the selection of these islands. Then, the “Scissors” tool is used.

**Figure 5:**
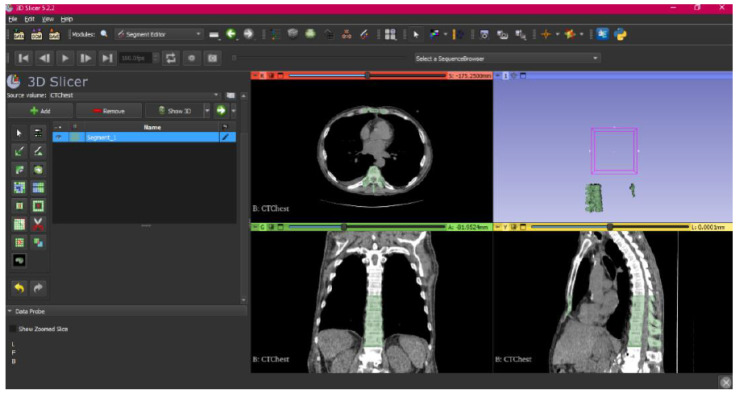
Segment Editor, “Scissors” tool.

The tool has allowed regions to be cut, which has served to eliminate unwanted parts of the segment. Next, the “Paint” tool is used.

The tool has allowed us to paint regions of interest in each of the views, which has served to select parts that have not been selected with the previous tools. Next, the “Erase” tool is used.

This tool has allowed us to delete regions of the segment from each of the views, which has served to eliminate unwanted parts. Next, the “Smoothing” tool is used.

**Figure 6:**
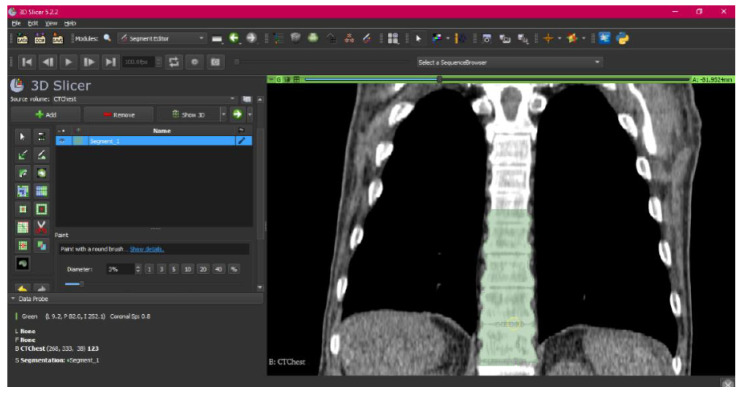
Segment Editor, “Paint” tool.

The tool has allowed specific regions to be smoothed in the section views, which has served to correct small segmentation errors in some areas while preserving all the details in other regions.

**Figure 7:**
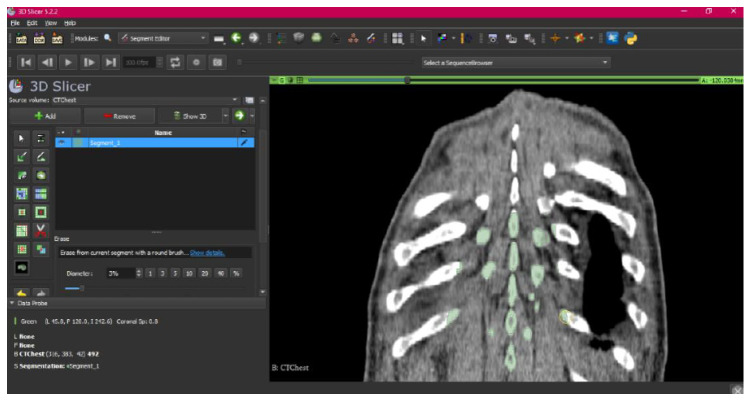
Segment Editor, “Erase” tool.

**Figure 8:**
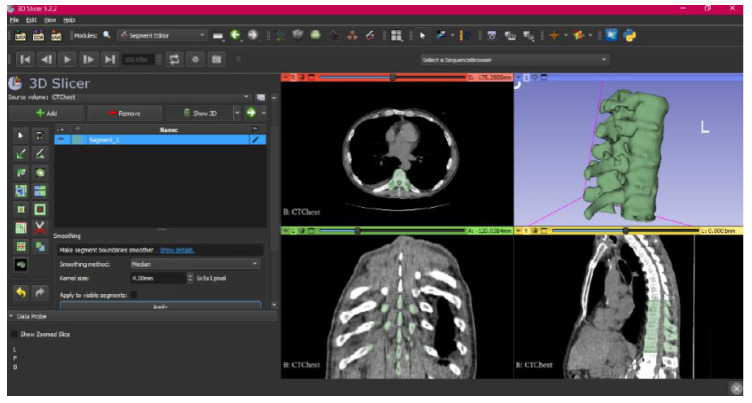
Segment Editor, “Smoothing” tool.

**Figure 9:**
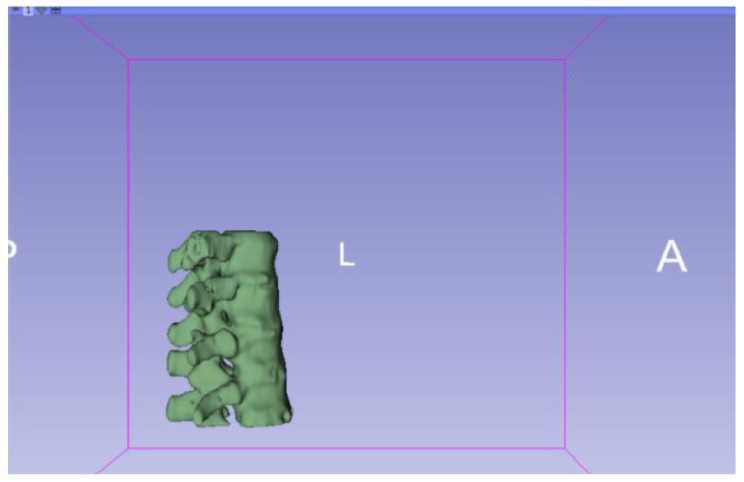
Three-dimensional visualisation window from the right part.

**Figure 10:**
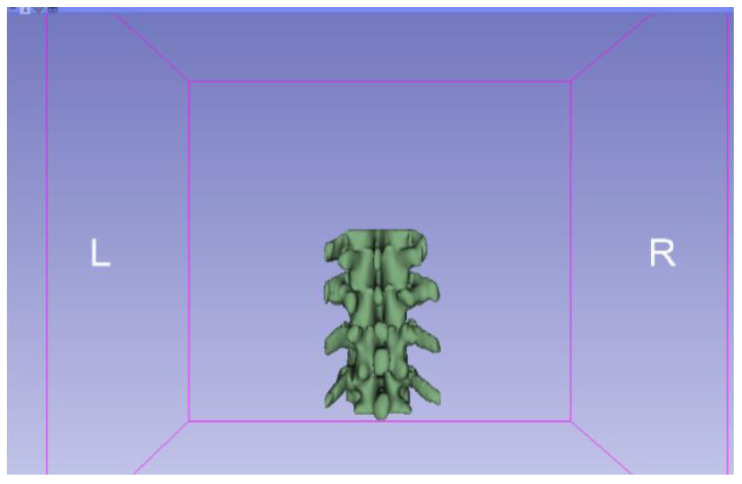
Three-dimensional visualisation window from the right part.

The outlined protocol spans from the import of DICOM files to the segmentation and refinement of three-dimensional models. Segmentation is a critical phase, as it necessitates the identification of relevant structures and the removal of undesired components. Throughout this process, thresholds and specific segmentation tools are utilised to delineate areas of interest, as has been previously demonstrated. Precision in segmentation is crucial for generating accurate 3D models that faithfully replicate anatomical features.

**Figure 11:**
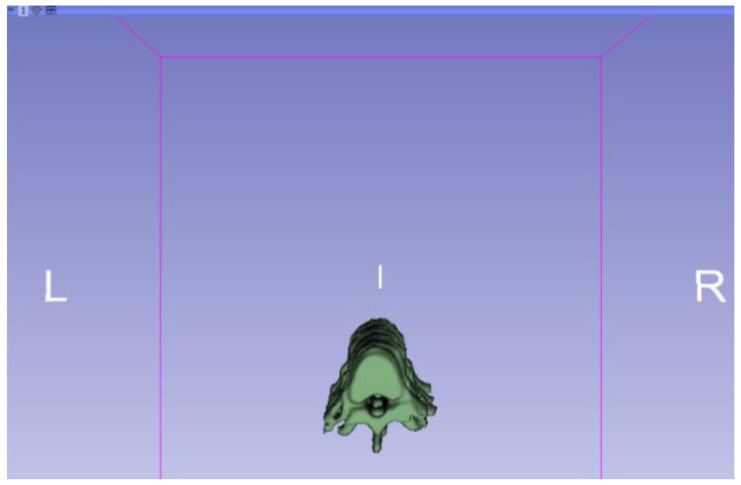
Three-dimensional visualisation window from the upper part.

**Figure 12:**
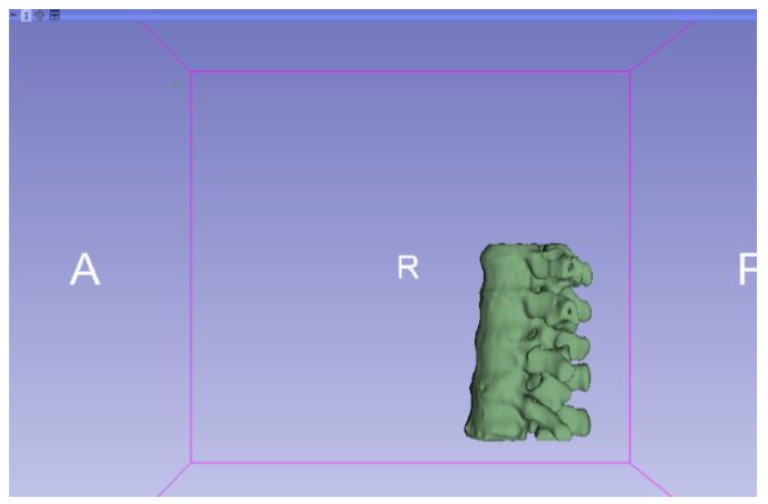
Three-dimensional visualisation window from the left part.

**Figure 13:**
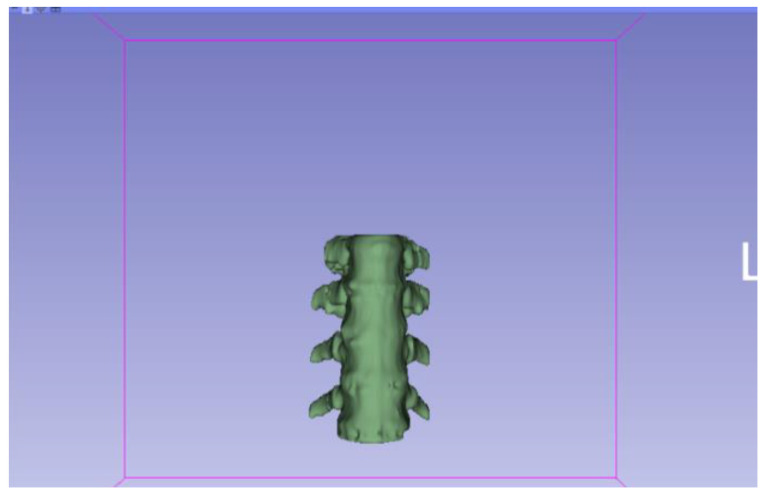
Three-dimensional visualisation window from the front part.

**Figure 14:**
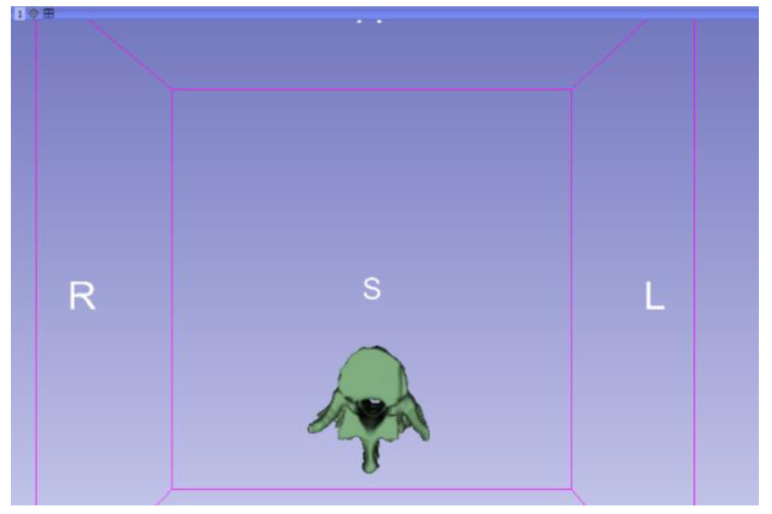
Three-dimensional visualisation window from the lower part.

#### 3.1.2. STL-to-GCODE File

Starting from the STL file generated in 3D Slicer, the second part of the protocol begins. Once all parameters are configured, Ultimaker Cura generates a “PREPARE” view.

**Figure 15:**
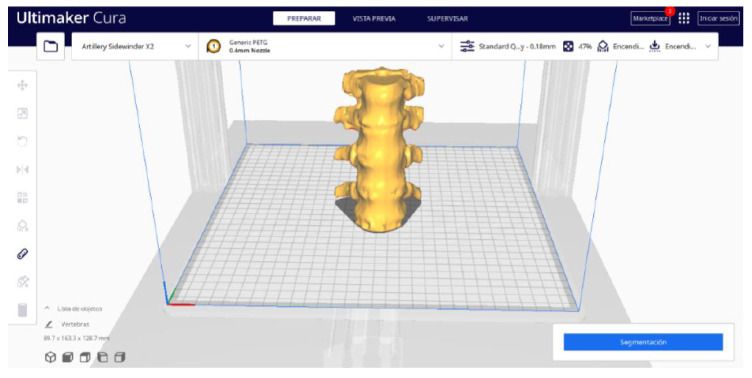
Visualisation 1 in “Prepare”.

**Figure 16:**
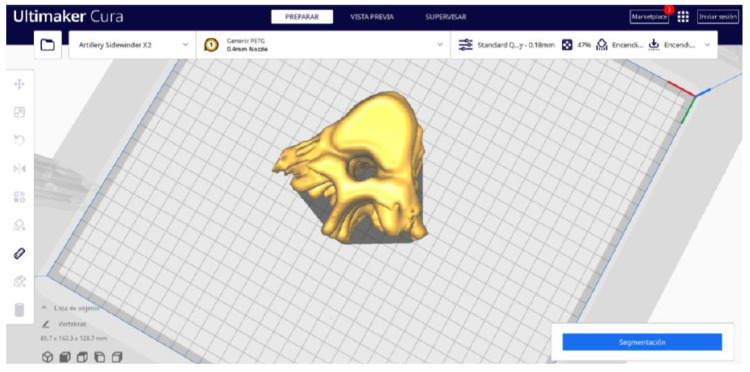
Visualisation 2 in “Prepare”.

**Figure 17:**
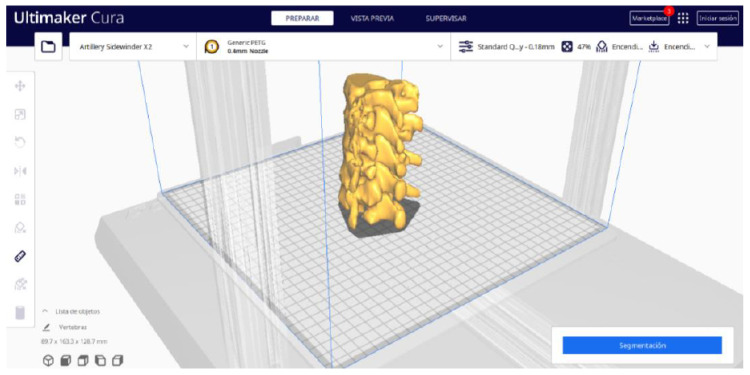
Visualisation 3 in “Prepare”.

**Figure 18:**
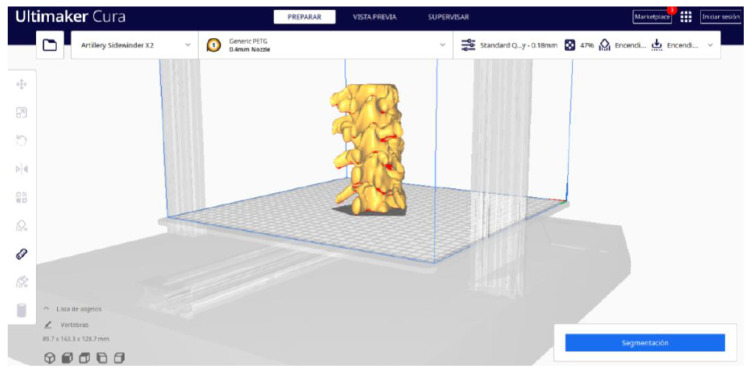
Visualisation 4 in “Prepare”.

From Figure 15, Figure 16, Figure 17 and Figure 18, it can be observed that the STL model accurately reflects the anatomical features, in this case, bone structures, present in the DICOM file.

**Figure 19:**
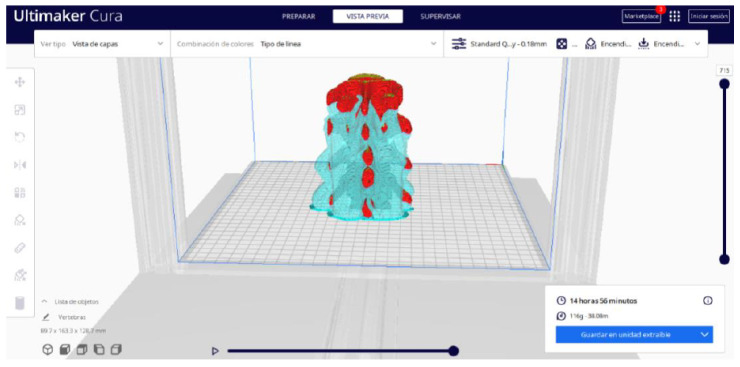
Visualisation 1 in “Preview”.

**Figure 20:**
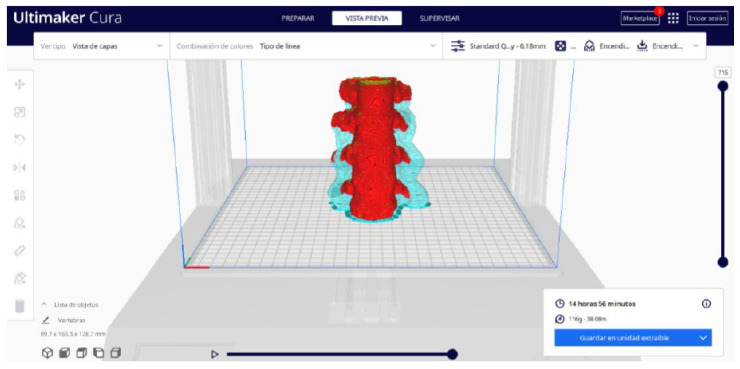
Visualisation 2 in “Preview”.

**Figure 21:**
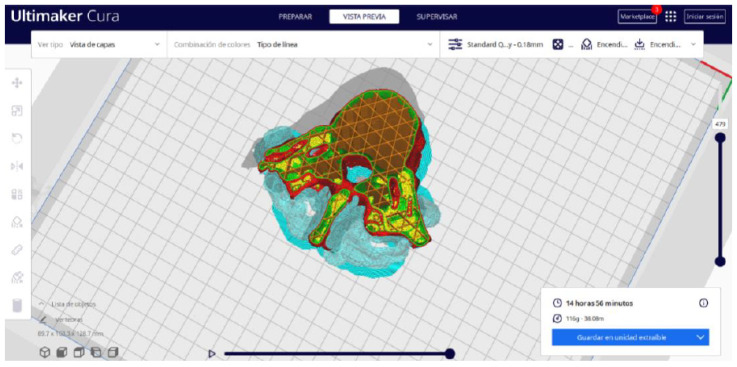
Visualisation 3 in “Preview”.

Ultimaker Cura also previews the 3D print in its “Layered View” viewing mode (Figure 19, Figure 20 and Figure 21). This view allows you to verify the path that the printer nozzle will follow during each layer of the print.

#### 3.1.3. Final Printing

The quality of 3D prints is largely dependent on the parameters set within the slicing software, such as Ultimaker Cura. Generally, a lower layer height, reduced speed, precise temperature adjustment, and the use of supports will result in a higher-quality figure. However, these settings can also lead to an increased printing time. Furthermore, the quality also hinges on how well the printer has been calibrated.

In this case, the print was produced at the original scale using the Artillery Sidewinder X2, and another print was made at a reduced scale of 26.16% with the Creality Ender 3 printer.

**Figure 22:**
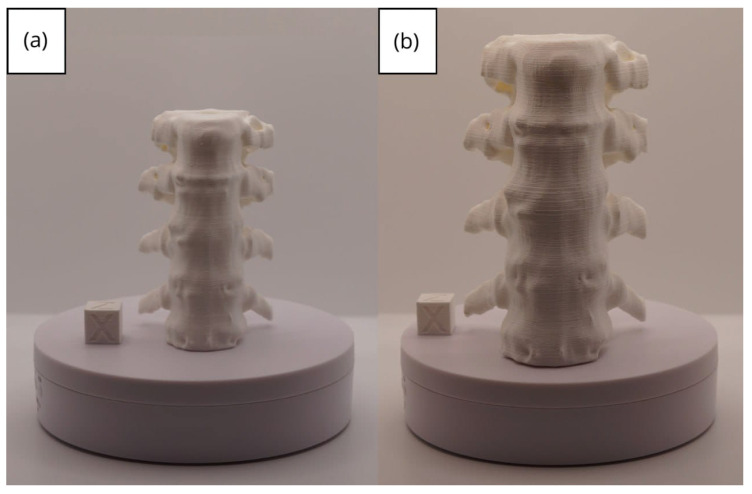
Front view of the vertebra: (**a**) Ender 3 printer, scaled figure; (**b**) Artillery Sidewinder X2 printer.

**Figure 23:**
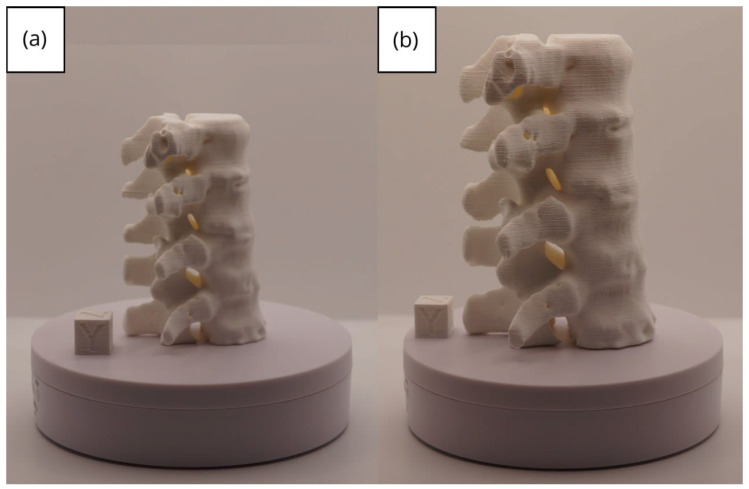
Left profile vertebra: (**a**) Ender 3 printer, scaled figure; (**b**) Artillery Sidewinder X2 printer.

**Figure 24:**
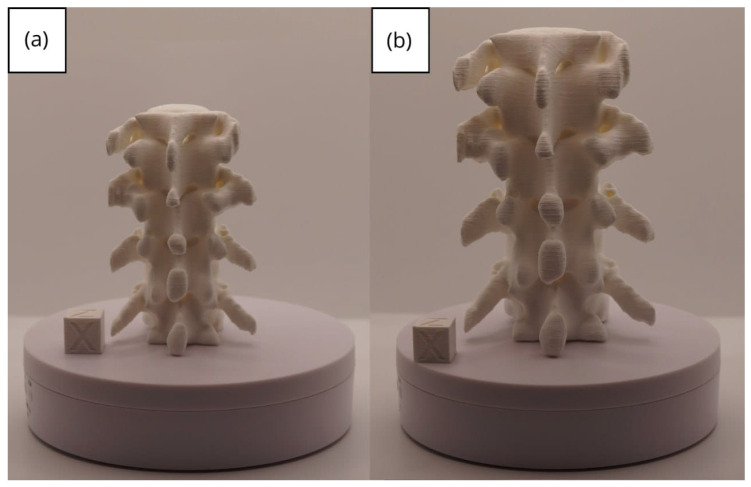
Posterior view vertebra: (**a**) Ender 3 printer, scaled figure; (**b**) Artillery Sidewinder X2 printer.

**Figure 25:**
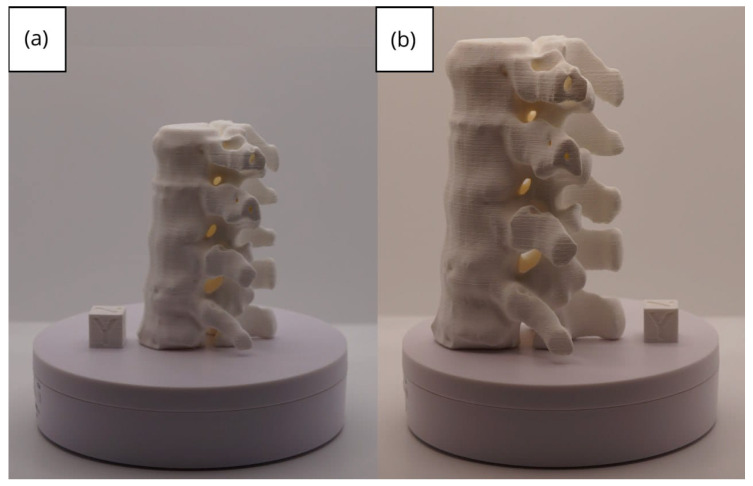
Right profile vertebra: (**a**) Ender 3 printer, scaled figure; (**b**) Artillery Sidewinder X2 printer.

**Figure 26:**
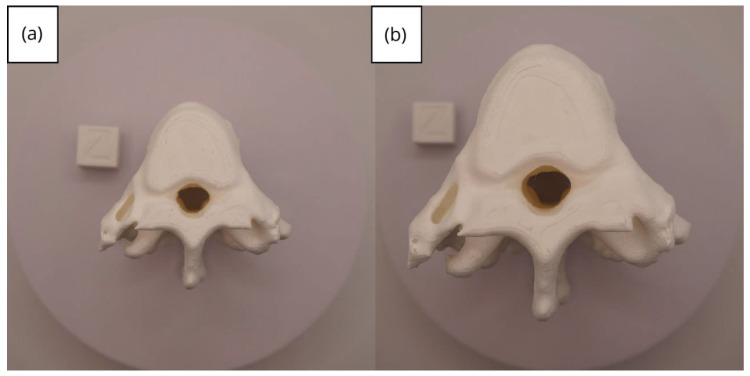
Upper view vertebra: (**a**) Ender 3 printer, scaled figure; (**b**) Artillery Sidewinder X2 printer.

From Figure 22, Figure 23, Figure 24, Figure 25 and Figure 26, it is observable that the prints exhibit a smooth and continuous surface, without significant defects, which confirms the fidelity of the 3D representation of the original anatomical structures in the DICOM files.

Standard surface roughness values (Ra and Rz) for each printed model, presented in tabular format, are given in Table 2 [22].

Printing technology fundamentally influences surface roughness in the following ways [23]: (1) Fused Deposition Modelling printers, as used in our study, typically operate with standard layer heights of 0.16–0.2 mm, which directly impacts surface texture. By comparison, (2) Resin-based technologies such as SLA or DLP can achieve significantly finer layer heights (standard 0.05 mm), resulting in smoother surfaces but with different material constraints.

As per the analysis of material considerations in relation to our protocol’s primary applications, the following should be considered: (1) For surgical planning and medical education—the principal applications of our protocol—we emphasise that material selection involves crucial trade-offs between surface finish, biocompatibility, and cost-effectiveness. (2) Resin-based prints (SLA/DLP) can achieve superior surface smoothness, but the materials typically involve higher chemical complexity and substantially greater cost. (3) PLA filament, particularly peroxide-sterilisable variants, offers several advantages for our intended applications, including cost-effectiveness, adequate surface quality for visualisation purposes, and compatibility with basic sterilisation procedures.

On the other hand, we would like to clarify that optimal surface roughness is not an absolute value but rather application-dependent, with different requirements for educational models, surgical planning templates, and patient-specific implants [24]. For surgical planning and educational applications, our protocol’s emphasis on accessibility and cost-effectiveness justifies the surface quality achieved with FDM technology. This expanded analysis provides the essential context for evaluating the quality of models produced using our protocol, acknowledging that surface roughness represents one of several interdependent parameters (including dimensional accuracy, material properties, and production cost) that must be balanced according to specific clinical and educational requirements.

### 3.2. Human Lower Jaw

#### 3.2.1. DICOM-to-STL Files

**Figure 27:**
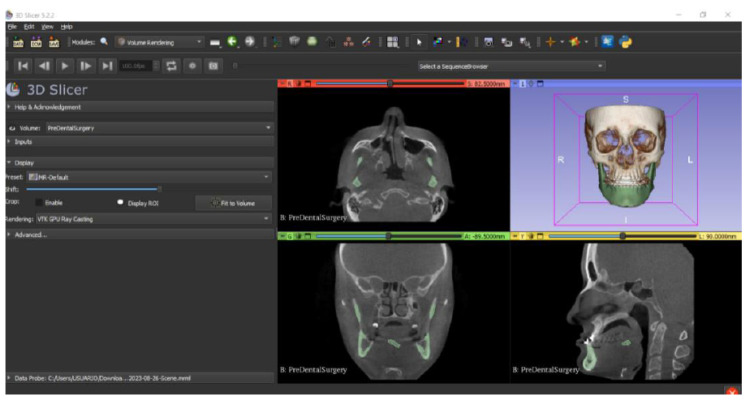
DICOM CBCTDentalSurgery Sample Data PreDentalSurgery in “Four-Up” configuration.

**Figure 28:**
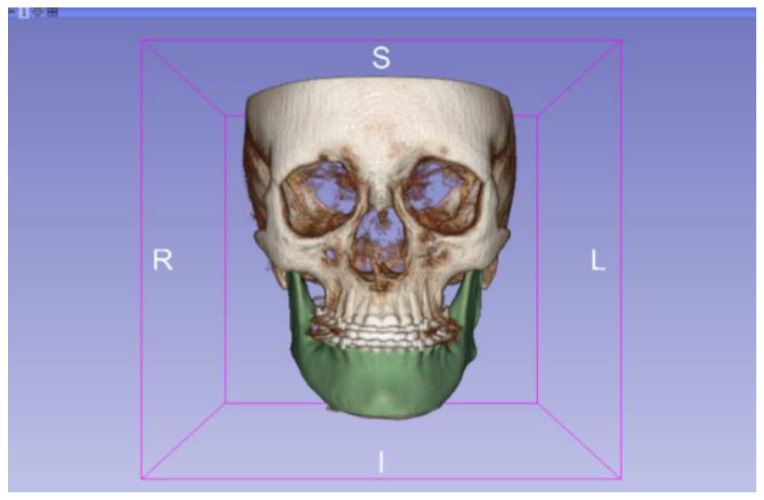
Three-dimensional visualisation window from the front part of the segmentation in relation to the computer tomography image.

**Figure 29:**
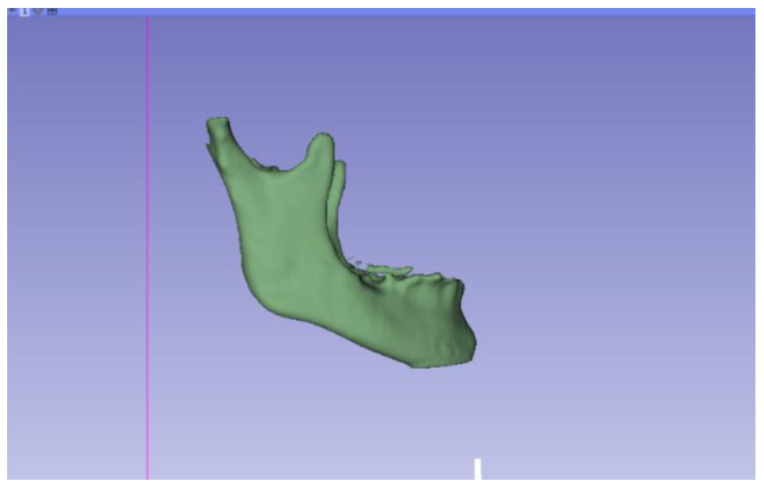
Three-dimensional visualisation window from the right part.

**Figure 30:**
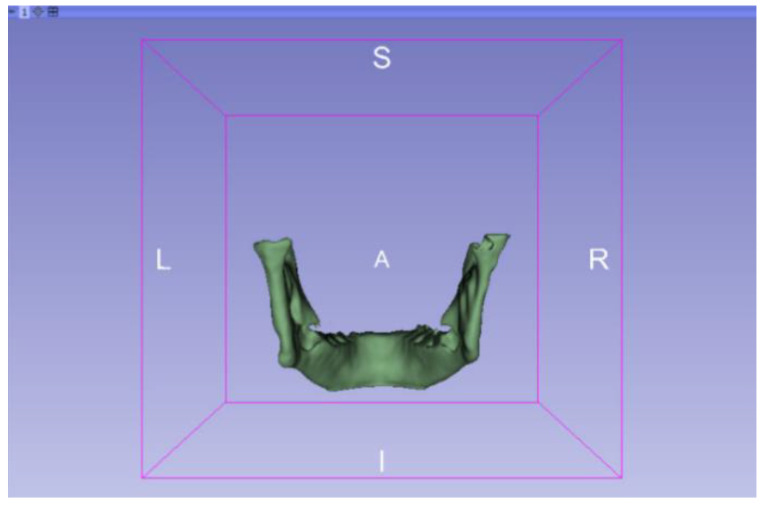
Three-dimensional visualisation window from the posterior part.

**Figure 31:**
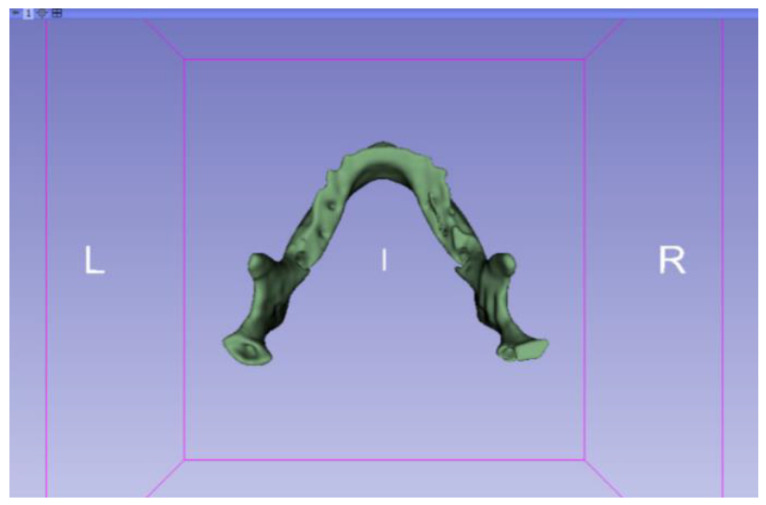
Three-dimensional visualisation window from the upper part.

**Figure 32:**
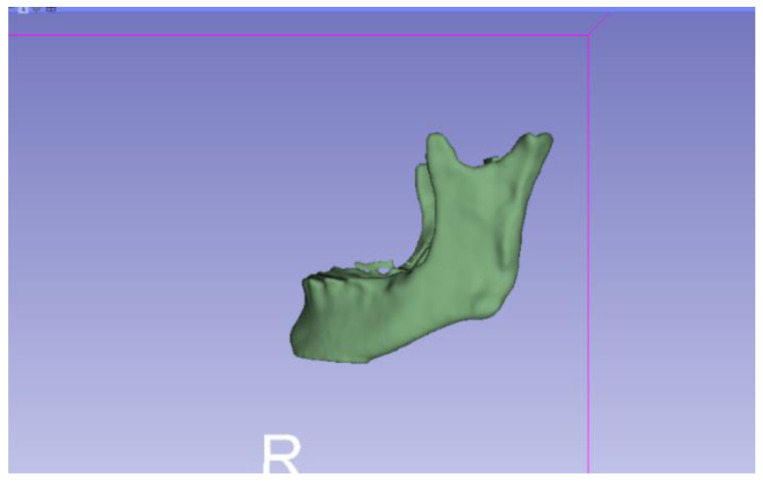
Three-dimensional visualisation window from the left part.

**Figure 33:**
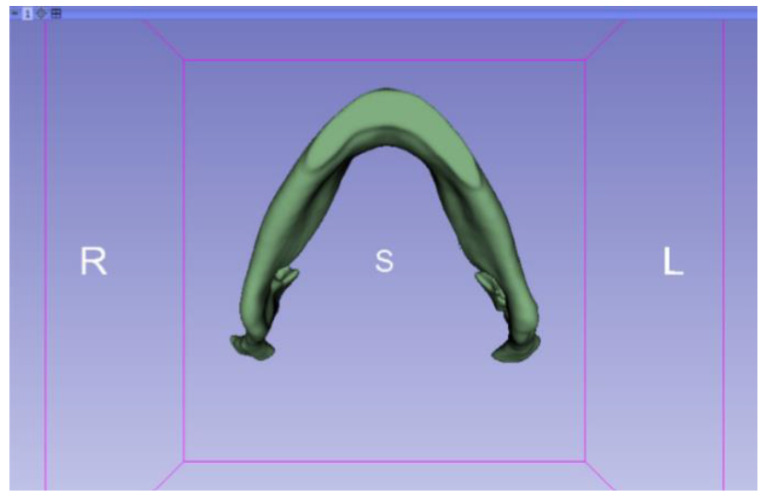
Three-dimensional visualisation window from the lower part.

In this particular case, the file called “CBCTDentalSurgery” (Cone Beam Computed Tomography) PreDentalSurgery was downloaded. The procedure is carried out through the series of steps present in Section 2.1, whose methodology is completely equivalent to the one applied to the vertebrae case (Figure 27, Figure 28, Figure 29, Figure 30, Figure 31, Figure 32 and Figure 33).

The fundamental difference in this case lies in the type of DICOM file selected. Unlike the previous example, which was based on a traditional Computed Tomography (CT), this time, a file from a Cone Beam Computed Tomography (CBCT) was chosen. This distinction does not alter the procedure for converting the DICOM file into a three-dimensional STL model, a process that remains identical to that previously described.

The implementation of this procedure gives an STL file as an output, which reflects the dental anatomy under study, facilitating a visualisation of the dental structure before the surgical intervention. This result emphasises the protocol ability to be versatile and adaptable to various imaging modalities, highlighting the usefulness of CBCT in the field of dental surgery.

Cone beam tomography, in particular, provides high-resolution images, which are important for detailed surgical planning and the comprehensive evaluation of dental anatomy [25].

#### 3.2.2. STL-to-GCODE Files

Beginning from the STL file generated through the use of 3D Slicer, the second stage of the protocol defined for this protocol is initiated. The procedure unfolds by following the sequence of steps detailed in Section 2.2.

**Figure 34:**
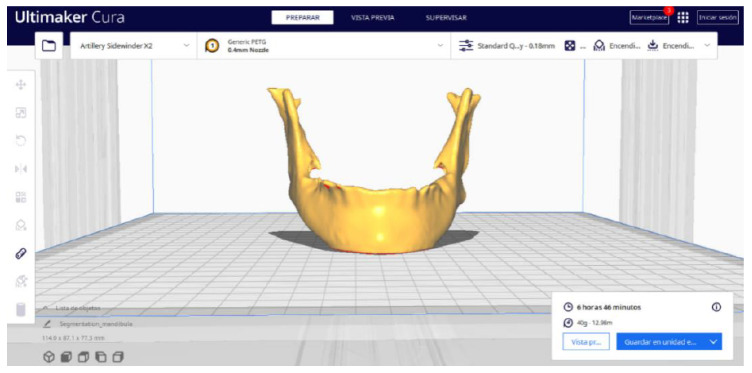
Visualisation 1 in “Prepare”.

**Figure 35:**
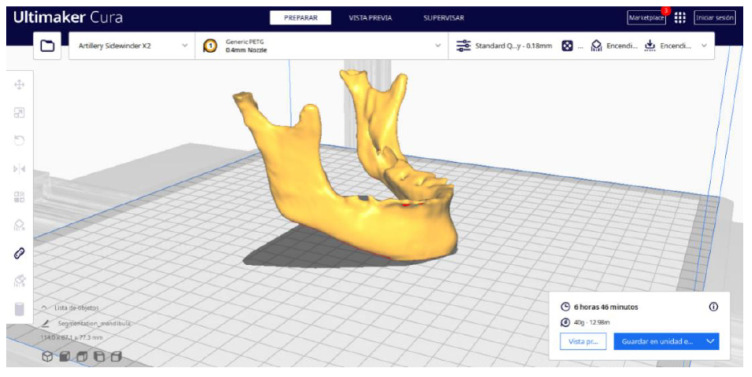
Visualisation 2 in “Prepare”.

**Figure 36:**
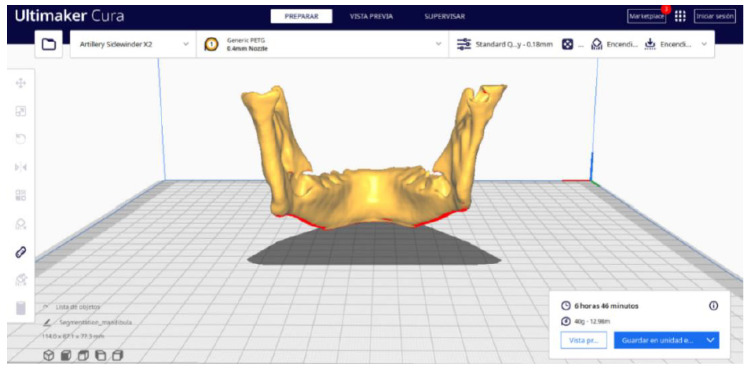
Visualisation 3 in “Prepare”.

**Figure 37:**
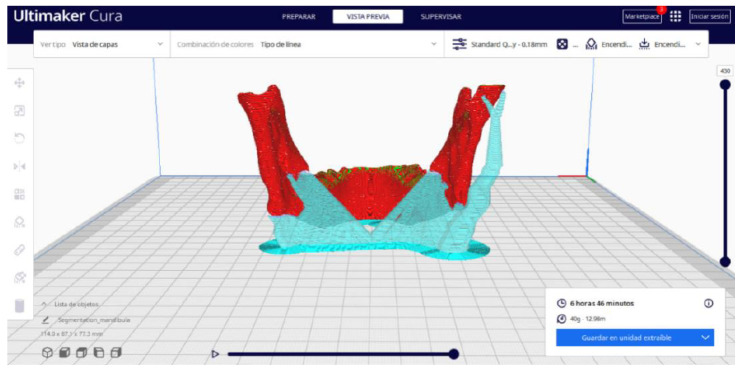
Visualisation 1 in “Preview”.

**Figure 38:**
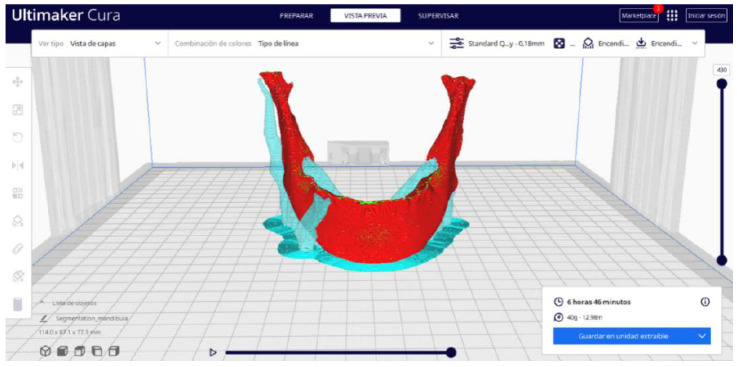
Visualisation 2 in “Preview”.

**Figure 39:**
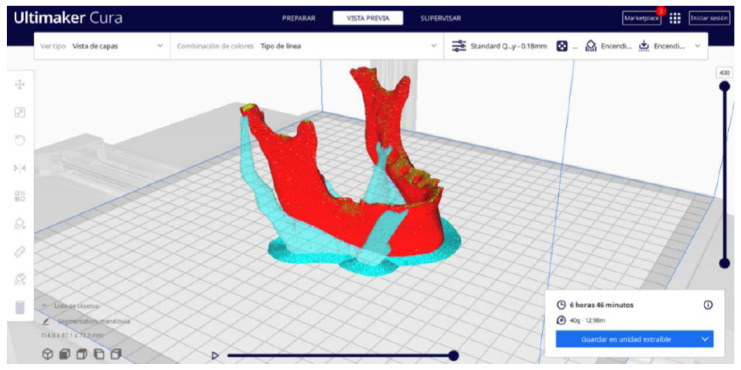
Visualisation 3 in “Preview”.

**Figure 40:**
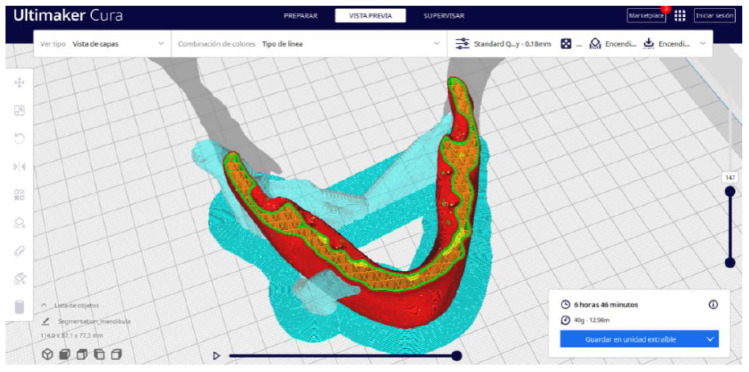
Visualisation 4 in “Preview”.

Upon examining Figure 34, Figure 35 and Figure 36, the STL model precisely captures the anatomical characteristics, specifically bone structures, depicted in the DICOM file. The Ultimaker Cura visualization in “Preview” mode is given in Figure 37, Figure 38, Figure 39 and Figure 40.

#### 3.2.3. Final Printing

**Figure 41:**
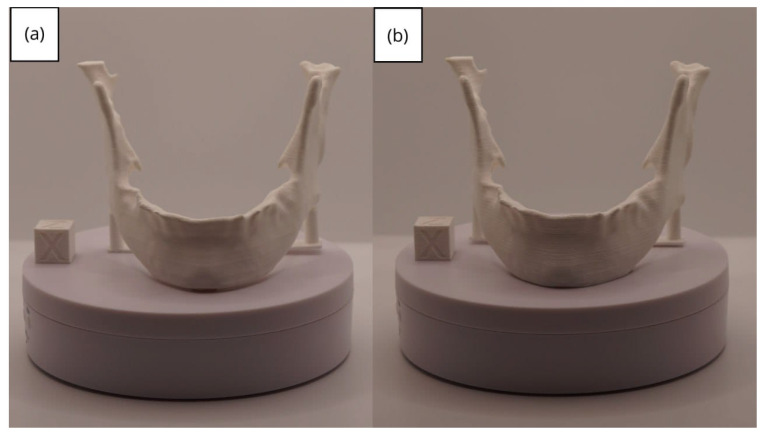
Front view of the lower jaw: (**a**) Ender 3 printer; (**b**) Artillery Sidewinder X2 printer.

**Figure 42:**
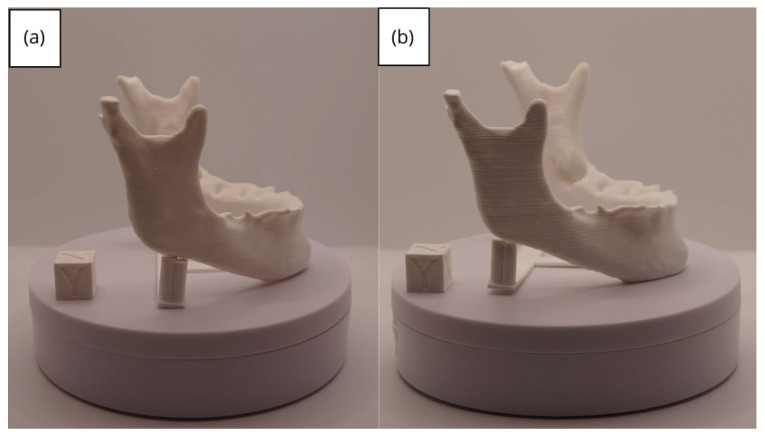
Left profile of the lower jaw: (**a**) Ender 3 printer; (**b**) Artillery Sidewinder X2 printer.

**Figure 43:**
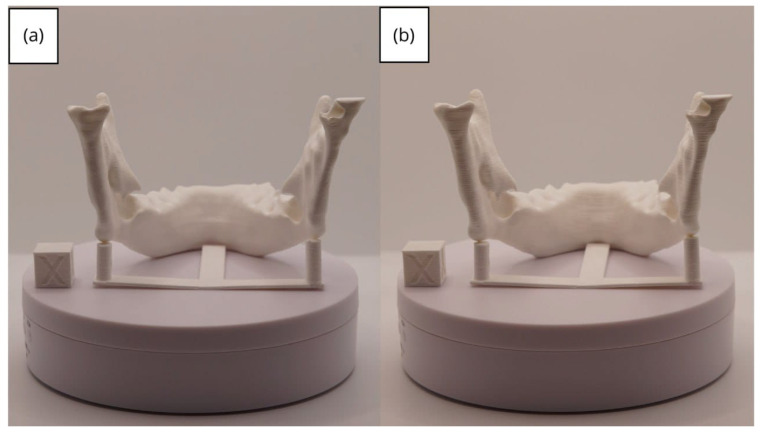
Posterior view of the lower jaw: (**a**) Ender 3 printer; (**b**) Artillery Sidewinder X2 printer.

**Figure 44:**
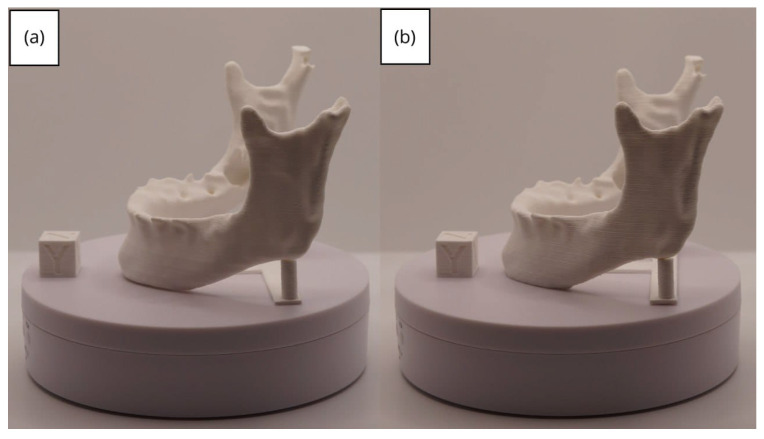
Right profile of the lower jaw: (**a**) Ender 3 printer; (**b**) Artillery Sidewinder X2 printer.

**Figure 45:**
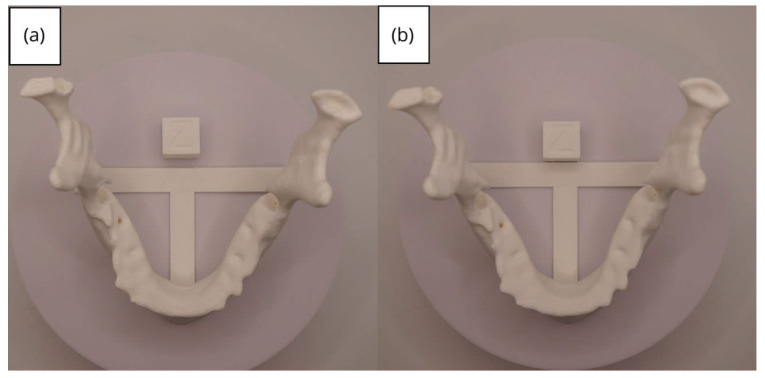
Upper view of the lower jaw: (**a**) Ender 3 printer; (**b**) Artillery Sidewinder X2 printer.

From Figure 41, Figure 42, Figure 43, Figure 44 and Figure 45, the prints exhibit a smooth and continuous surface, free from significant defects, underscoring the accuracy of the 3D representation of the original anatomical structures contained within the DICOM files.

### 3.3. Human Brain Tumour

#### 3.3.1. DICOM-to-STL Files

**Figure 46:**
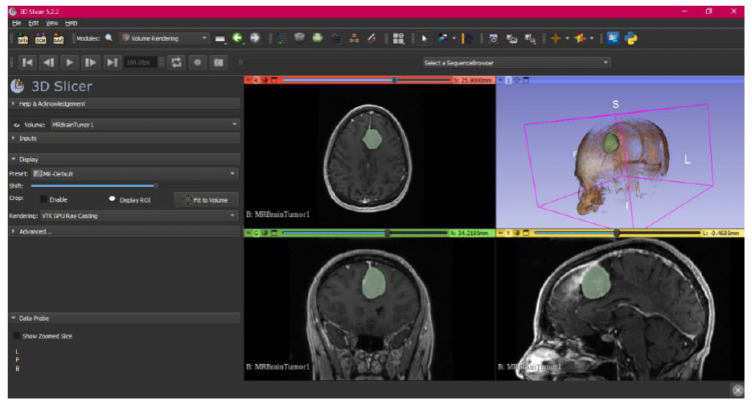
DICOM MRBrainTumor1 Sample Data in “Four-Up” configuration.

**Figure 47:**
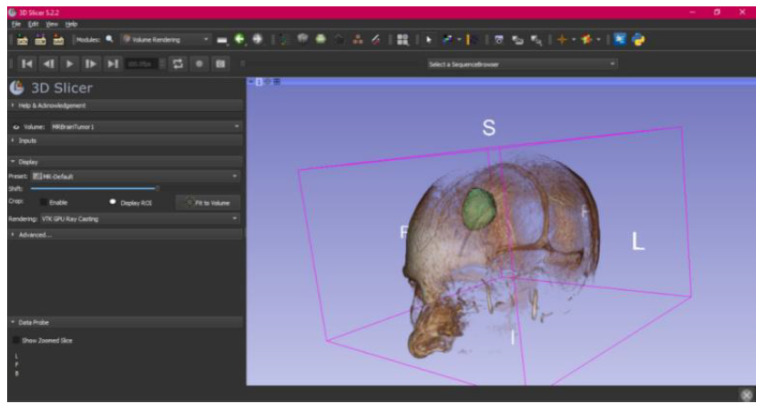
Three-dimensional window visualisation 1 of the segmentation in relation to the magnetic resonance.

**Figure 48:**
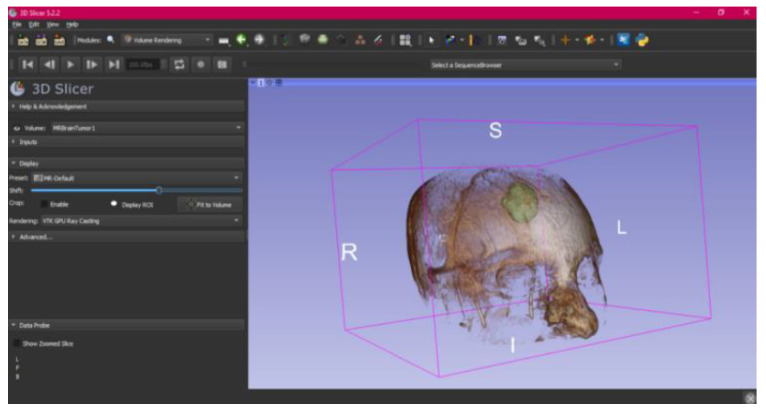
Three-dimensional window visualisation 2 of the segmentation in relation to the magnetic resonance.

**Figure 49:**
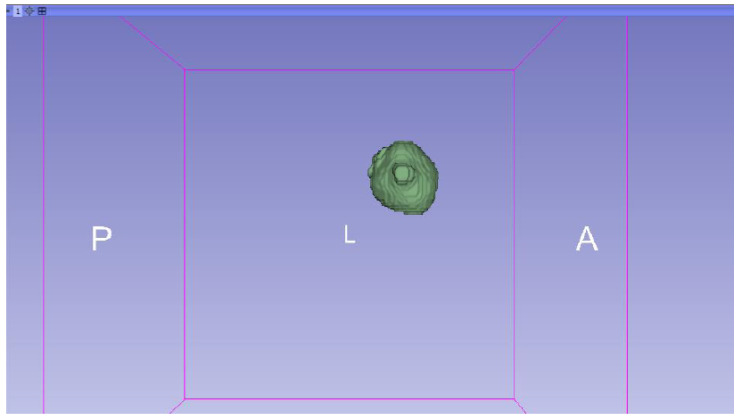
Three-dimensional visualisation window from the right part.

**Figure 50:**
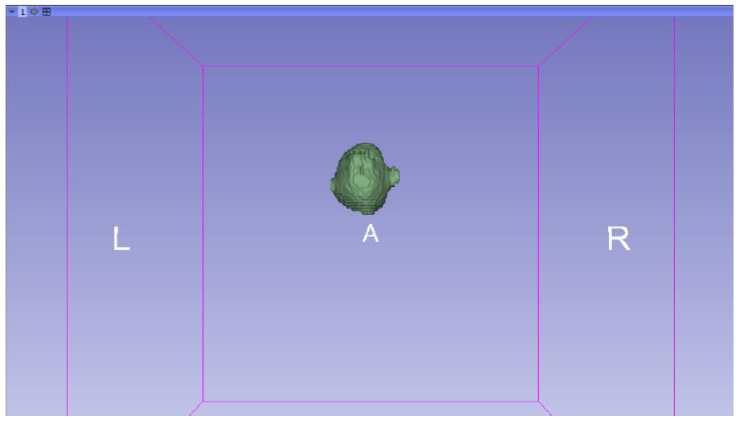
Three-dimensional visualisation window from the posterior part.

**Figure 51:**
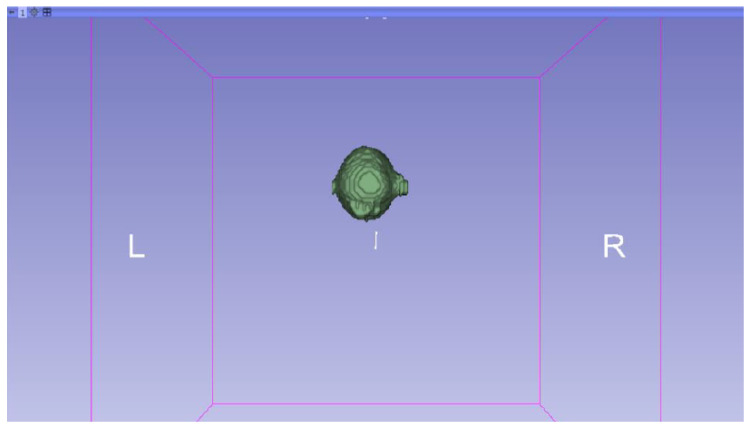
Three-dimensional visualisation window from the superior part.

**Figure 52:**
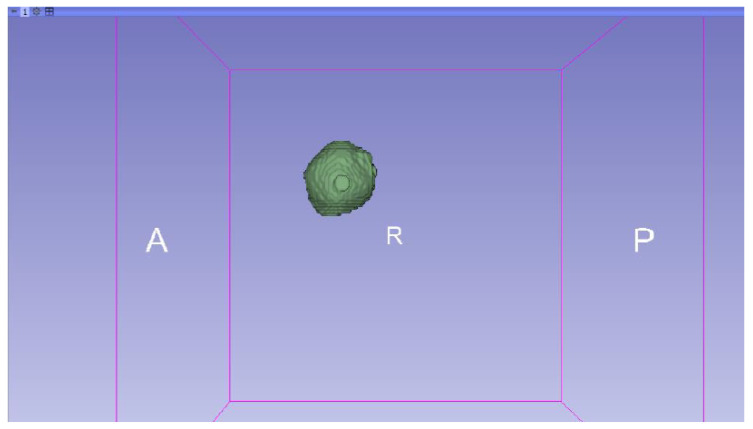
Three-dimensional visualisation window from the left part.

**Figure 53:**
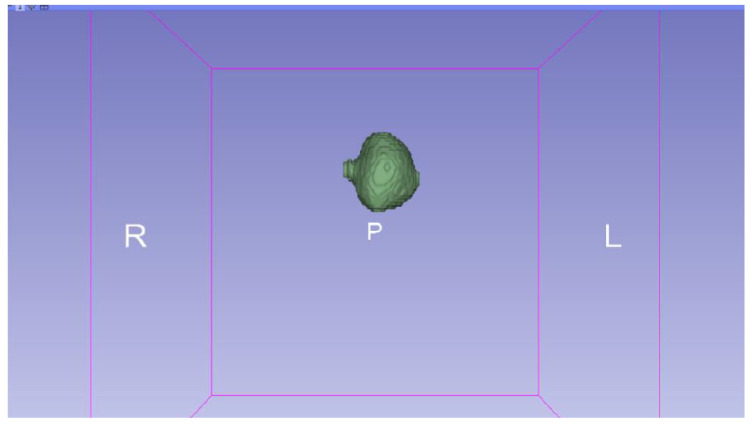
Three-dimensional visualisation window from the front part.

**Figure 54:**
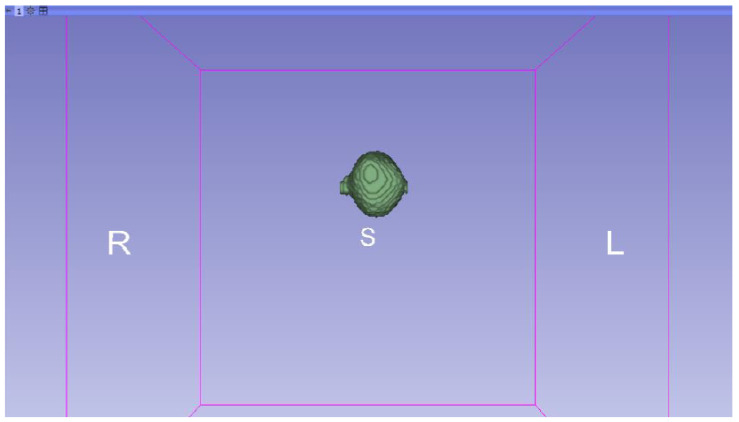
Three-dimensional visualisation window from the lower part.

In this specific instance, the file named “MRBrainTumor1” was obtained for analysis. The processing of this file was conducted in accordance with the series of steps outlined in Section 2.1 (Figure 46, Figure 47, Figure 48, Figure 49, Figure 50, Figure 51, Figure 52, Figure 53 and Figure 54). It is important to note that the methodology employed here is identical to that used in the vertebral analysis.

The fundamental distinction in this case lies in the selection of the DICOM file type, similarly to the previous case. Unlike the earlier examples, which were based on Computed Tomography (CT) scans, in this scenario, a file derived from Magnetic Resonance (MR) imaging was selected. This differentiation does not modify the established procedure for converting the DICOM file into a three-dimensional STL model. The conversion process remains unchanged and follows the methodology outlined in the previous description.

The implementation of this procedure results in an STL file, which accurately reflects the brain tumour under investigation and facilitates detailed visualisation. This outcome underscores the protocol’s versatility and adaptability across various imaging techniques, highlighting the crucial role of Magnetic Resonance Imaging (MRI) within the healthcare domain. Specifically, MRI provides high-resolution images that are critical for meticulous surgical planning and the comprehensive assessment of the tumour’s anatomical and pathological characteristics. This level of detail is essential for the precise evaluation of the tumour’s size, its location, and the potential involvement of surrounding tissues, which is paramount in determining the optimal surgical approach and minimising the risk of complications. Furthermore, MRI’s ability to distinguish between different types of tissues with great precision aids in the formulation of a more targeted treatment plan, thereby enhancing the effectiveness of therapeutic interventions.

#### 3.3.2. STL-to-GCODE Files

Starting from the STL file obtained through the use of 3D Slicer, the second phase of the protocol established for this study commences. This process proceeds according to the series of steps outlined in Section 2.2.

**Figure 55:**
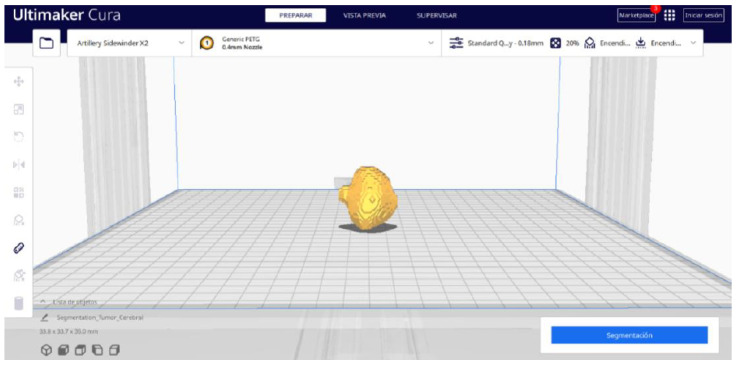
Visualisation 1 in “Prepare”.

**Figure 56:**
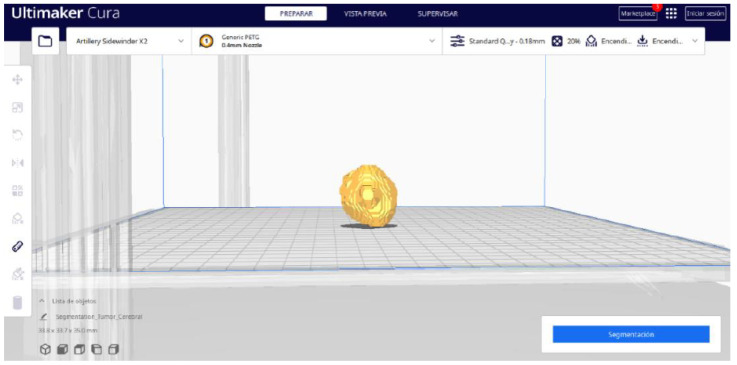
Visualisation 2 in “Prepare”.

**Figure 57:**
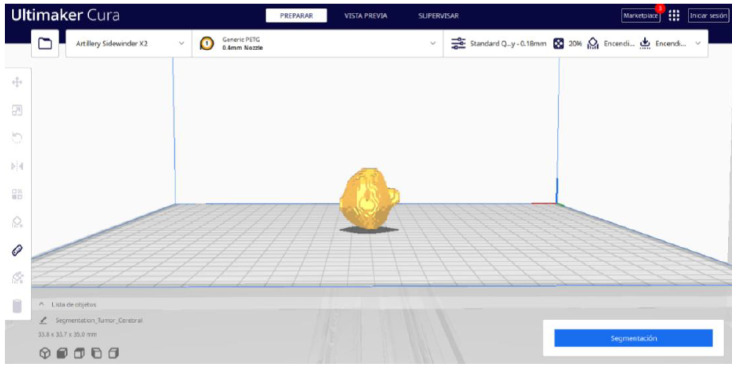
Visualisation 3 in “Prepare”.

**Figure 58:**
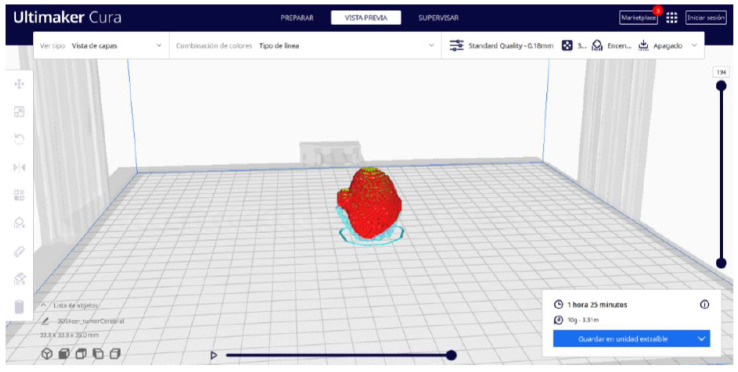
Visualisation 1 in “Preview”.

**Figure 59:**
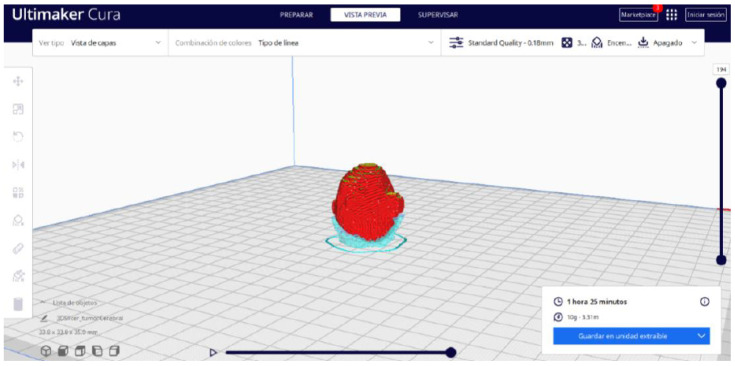
Visualisation 2 in “Preview”.

**Figure 60:**
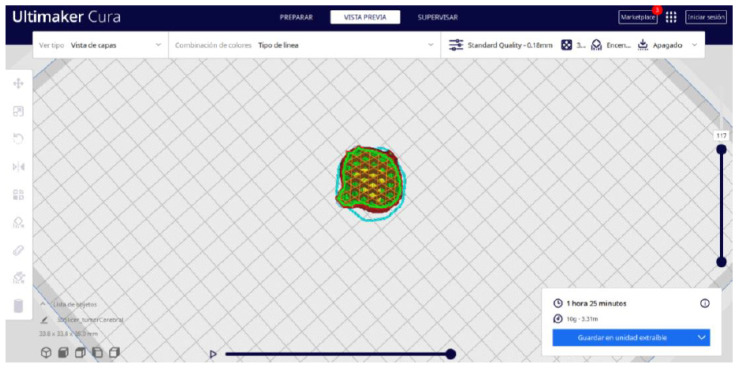
Visualisation 3 in “Preview”.

From Figure 55, Figure 56 and Figure 57, it is observable that the STL model accurately represents the anatomical features, in this instance, soft tissue, present in the DICOM file. It is important to note that the outcome of the brain tumour figure differs from those of the jaw and vertebrae. This difference is attributed to the MRI of the brain tumour exhibiting a greater distance between slices. Consequently, in the 3D print of the generated STL file, the height of the layers is visible. This may result in a slightly different appearance compared with the other figures. On the other hand, the visualization in “Preview” mode can be seen in Figure 58, Figure 59 and Figure 60.

#### 3.3.3. Final Printing

**Figure 61:**
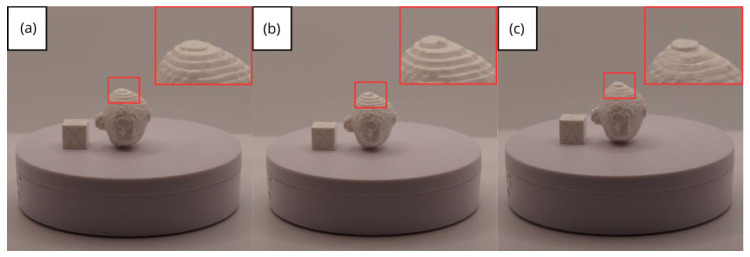
Front view of the brain tumour: (**a**) Ender 3 printer; (**b**) same as (**c**) printed upside-down; (**c**) Artillery Sidewinder X2 printer.

In addition to the two prints made with the two different printers, as in the previous cases, this time, an additional print was performed, printing in such a way that the piece was rotated 180 degrees on the y-axis. As can be seen in Figure 61, and more closely in the zoom, Figure 61b, it exhibits superior quality compared with the other printed pieces, showing greater definition.

**Figure 62:**
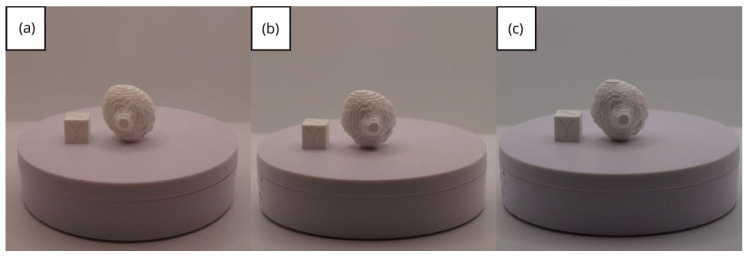
Left profile of the brain tumour: (**a**) Ender 3 printer; (**b**) same as (**c**) printed upside-down; (**c**) Artillery Sidewinder X2 printer.

**Figure 63:**
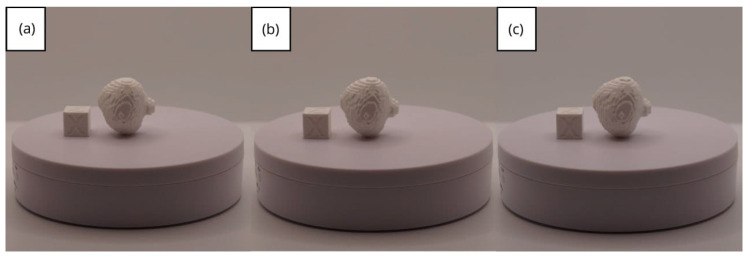
Posterior view of the brain tumour: (**a**) Ender 3 printer; (**b**) same as (**c**) printed upside-down; (**c**) Artillery Sidewinder X2 printer.

**Figure 64:**
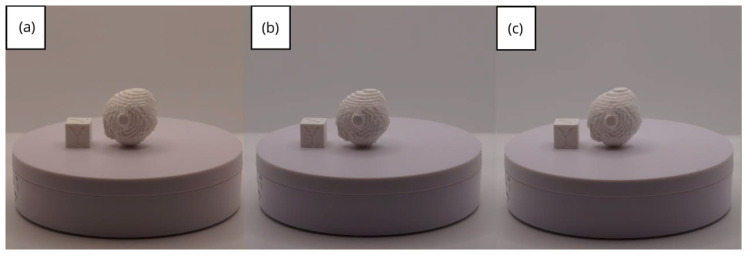
Right profile of the brain tumour: (**a**) Ender 3 printer; (**b**) same as (**c**) printed upside-down; (**c**) Artillery Sidewinder X2 printer.

**Figure 65:**
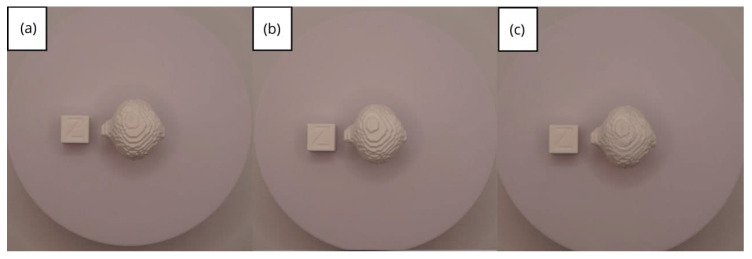
Upper view of the brain tumour: (**a**) Ender 3 printer; (**b**) same as (**c**) printed upside-down; (**c**) Artillery Sidewinder X2 printer.

From Figure 61, Figure 62, Figure 63, Figure 64 and Figure 65, the prints showcase a smooth and uninterrupted surface, without notable imperfections, confirming the fidelity of the 3D representation of the original anatomical structures in the DICOM files.

To evaluate the accuracy of the STL models, a comparison was conducted between the segmented 3D models and the original DICOM data. Minimal volumetric deviation was observed across the analysed cases, confirming high segmentation fidelity. Print resolution varied with model complexity, employing a layer height that captured fine anatomical details in all instances.

Compared with existing DICOM-to-STL workflows, our protocol demonstrates similar segmentation accuracy while significantly reducing costs and software dependency. Traditional methods relying on proprietary software may achieve slightly lower segmentation deviations (∼[1, 2]%), but at the expense of increased licensing costs and processing time. Potential sources of error include the over-segmentation of low-contrast regions in MRI-derived models and layer interpolation inaccuracies in high-curvature structures. Future refinements integrating AI-assisted segmentation could further enhance accuracy while maintaining workflow efficiency.

Reproducibility is a key factor in medical 3D printing workflows. While the protocol was applied consistently across different anatomical structures (vertebrae, jawbone, brain tumour), a formal inter-operator variability study was not conducted within the scope of this work. However, segmentation settings and STL processing parameters were standardised, leading to highly consistent results across independent trials. Future work will focus on a multi-operator validation study, where multiple users with varying experience levels will follow the protocol to assess differences in segmentation precision, processing time, and final model fidelity. Such an analysis will further reinforce the reliability of this methodology in clinical and research environments.

In order to corroborate the accuracy and reliability of our DICOM-to-STL conversion protocol, we performed a series of validation experiments on real segmented models, focusing on determining segmentation accuracy, processing efficiency, and print quality.

We evaluated the accuracy of our segmentation process by comparing the generated 3D models with the original DICOM datasets of real patients. Metrics such as the Dice Similarity Coefficient (DSC) and volumetric superposition were employed to quantify the agreement between the segmented results and reference images. Our findings indicate a high degree of agreement, reflecting the robustness of the method in accurately capturing anatomical structures.

The fidelity of the 3D printed models was examined by examining key attributes such as surface smoothness, dimensional accuracy, and structural integrity. These evaluations carried out by medical personnel confirm that the printed models maintain the quality standards necessary for their effective use in medical contexts. The models used for these validation processes were carried out on real patient models, which, for confidentiality reasons, are not shown in this paper.

Finally, the main limitations for our methodology would be as follows:

1. **Resolution loss:** We can see in our protocol how the conversion process can lead to a reduction in image resolution, which could affect the fidelity of the 3D models. It is important to consider defining strategies in the DICOM file acquisition process to mitigate this resolution loss problem, such as optimising imaging parameters and carefully selecting segmentation thresholds. These losses are primarily dependent on the imaging process and are, therefore, beyond our control and the development of this protocol.
2. **Segmentation errors:** The potential for inaccuracies to occur during the segmentation phase is acknowledged, with emphasis placed on the difficulties in distinguishing between tissues of similar densities. We emphasise the importance of operator experience and the use of advanced segmentation tools to minimise these errors.
3. **Software limitations:** We examine the limitations associated with the open-source software used in our workflow, including processing capabilities and compatibility issues. Suggestions are provided to overcome these limitations, such as hardware upgrades and alternative software options.

## 4. Conclusions and Final Work

This study has effectively established the importance and feasibility of a comprehensive protocol for the conversion of DICOM files to STL models and their subsequent preparation for 3D printing by conversion to GCODE. This approach ensures the creation of three-dimensional models from medical images. The conversion of DICOM files to STL has been identified as a crucial step in generating accurate three-dimensional representations of anatomical and pathological structures. This would be a useful tool in a personalised medicine model that is increasingly closer to the objectives that we can simultaneously find in those defined in Industry 4.0 and digitalisation processes.

However, some limitations were observed in the conversion process. These include artifacts in the original DICOM images that can affect the quality of the STL models, such as blurred areas, image noise, and contrast anomalies. To mitigate these issues, preprocessing techniques can be applied before segmentation. Gaussian smoothing helps reduce high-frequency noise, while bilateral filtering preserves edges while minimising artifacts. Histogram equalisation can enhance contrast in low-visibility regions, improving segmentation accuracy. Implementing these preprocessing steps can significantly enhance the fidelity of the final 3D model, particularly with complex anatomical structures.

When converting DICOM files to STL models, the presence of artifacts in the original images can affect the quality and accuracy of the generated 3D models since these models are based on the information contained in the images. Therefore, the presence of artifacts can introduce inaccuracies in the resulting STL model.

To address these limitations, it is important to consider the possibility of artifacts in DICOM images and to make appropriate adjustments to the thresholding and segmentation parameters during the conversion process to minimise their impact on the final quality of the STL model.

It is also important to consider the conditions under which each of the medical diagnostic tests are performed. Factors such as the distance taken for each slice, specific exam conditions, and patient movements can influence the quality of the resulting DICOM files.

If the DICOM file you are working with is of high quality, a better-quality STL file will be obtained. However, this represents a limitation since, if the quality of the DICOM file is low, it cannot be modified, and, as a result, the STL files will have lower quality. This is an important consideration to keep in mind when working with these types of files in the medical field.

Future lines of work would include: (a) As the follow-up main research line of the work carried out in this research, we would aim at evolving the DICOM-to-STL protocol, following the evolution of FDM printing machines. In the last half-year, high-speed multi-material machines have emerged on the market, which would not only allow for printing the segmented part of a DICOM file, but also allow for the use of different materials and colours, making it easier to give context to the main part of the segmented model, providing the necessary anatomical environment for an improvement in interpretation by the medical specialist. This is why the improvement in multi-material/multi-colour STL files will be the next objective in the short and medium term as a continuation of this research. (b) STL file generation through AI from a DICOM file is another point that we aim at exploring with a broader time horizon, given the emergence of large language models, or LLMs (Large Language Models), and other ML (Machine Learning) and DL tools, which allow us to improve the process of segmentation through natural language. This process would substantially accelerate the generation of STL files.

These two lines of work, even though they run in parallel and with different temporal spaces, allow for the continuation of the work carried out in this research.

On the other hand, future research should explore the integration of AI-driven segmentation techniques into the workflow to improve automation and segmentation precision, particularly for complex structures like cranial-maxillofacial models or tumour delineation [12]. Additionally, expanding the protocol to support multi-material and multi-colour 3D printing could further enhance the utility of patient-specific anatomical models, providing additional context for medical professionals.

For first-time users, we recommend starting with small and well-defined anatomical structures, such as vertebrae or single bone segments, before attempting complex models. Adjusting segmentation parameters iteratively, using preprocessing filters to enhance image quality, and validating STL outputs in a 3D viewer before printing can help optimise the results. Additionally, selecting appropriate print settings—such as [20,40]% infill density for rigid structures and a higher wall thickness for delicate models—ensures better print stability and anatomical accuracy.

## Figures and Tables

**Table 1:** Key parameters. ST: Segmentation Threshold; SK: Smoothing Kernel; VS: Voxel Size; LH: Layer Height; ID: Infill Density.

Case Study	ST (HU)	SK	VS (mm)	LH (mm)	ID (%)
Vertebrae	[150, 300]	Median (3×3)	0.8	0.18	25
Jawbone	[100, 250]	Gaussian (2×2)	0.6	0.20	40
Brain Tumour	[90, 220]	None	0.8	0.20	25

**Table 2:** Standard surface roughness values (Ra and Rz).

Model	Data (Ra/Rz)	Printer
Vertebrae models	Ra=12.7±1.5μm; Rz=87.4±8.2μm	Artillery Sidewinder X2
	Ra=18.3±2.1μm; Rz=105.6±10.5μm	Creality Ender 3
Lower jaw models	Ra=14.2±1.8μm; Rz=92.5±9.1μm	Artillery Sidewinder X2
	Ra=20.1±2.4μm; Rz=112.3±11.8μm	Creality Ender 3
Brain tumour models	Ra=15.8±1.9μm; Rz=98.7±9.5μm	Artillery Sidewinder X2
	Ra=22.5±2.7μm; Rz=118.9±12.3μm	Creality Ender 3

## Data Availability

Access to data will be granted on a reasonable basis. High-resolution images of Section 3.1.3, Section 3.2.3 and Section 3.3.3, as well as the STL files of each of the analysed DICOMs, can be found in the following GitHub repository. The datasets analysed during the current study are publicly available within the “3D Slicer” software, which can be accessed freely by users for further analysis and verification. Access date: 20 December 2024.

## Supplementary Materials

The printing parameters can be found in the Supplementary Material Videos of the printed figures, which can be found at the following links (accessed 20 December 2024): https://youtube.com/shorts/M7_woQqG0YI, https://youtube.com/shorts/kHb_YmN5jxA, https://youtube.com/shorts/ZIcetC74Nqs, https://youtube.com/shorts/5pEY3IuwZtE, https://youtube.com/shorts/1SgNUPt-hK0, https://youtube.com/shorts/bWfu-ugvJqM, https://youtube.com/shorts/VI6tc39xBs8.
